# Domiciliary transcutaneous electrical stimulation in patients with obstructive sleep apnoea and limited adherence to continuous positive airway pressure therapy: a single-centre, open-label, randomised, controlled phase III trial

**DOI:** 10.1016/j.eclinm.2023.102112

**Published:** 2023-08-03

**Authors:** Deeban Ratneswaran, Michael Cheng, Ebrahim Nasser, Rajiv Madula, Martino Pengo, Kath Hope, Esther I. Schwarz, Yuanming Luo, Georgios Kaltsakas, Michael I. Polkey, John Moxham, Joerg Steier

**Affiliations:** a Faculty of Life Sciences and Medicine, King’s College London, Centre for Human & Applied Physiological Sciences, London, UK; b Lane Fox Unit/Sleep Disorders Centre, Guy’s & St Thomas’ NHS Foundation Trust, London, UK; c Istituto Auxologico Italiano IRCCS, University of Milan, Milan, Italy; d Hope2Sleep Patient Charity, Hull, UK; e Department of Pulmonology, University Hospital Zurich and University of Zurich, Zurich, Switzerland; f State Key Laboratory of Respiratory Disease, Guangzhou Medical University, Guangzhou, China; g Royal Brompton & Harefield Campus, Guy’s & St Thomas’ NHS Foundation Trust, London, UK

**Keywords:** Genioglossus, CPAP, Non-CPAP therapy, Hypoglossal nerve stimulation

## Abstract

**Background:**

Hypoglossal nerve stimulation (HNS) for obstructive sleep apnoea (OSA) is a novel way to manage the condition. We hypothesised that in patients with OSA and limited adherence to continuous positive airway pressure (CPAP) therapy, domiciliary transcutaneous electrical stimulation (TESLA) would control sleep apnoea and provide health benefits.

**Methods:**

We undertook a single-centre, open-label, randomised, controlled phase III trial in patients with OSA (apnoea-hypopnoea-index [AHI] 5–35 h^−1^), a BMI of 18.5–32 kg∗m^−2^, and a documented lack of adherence to CPAP therapy (<4 h∗night^−1^) at Guy’s & St Thomas’ NHS Foundation Trust (hospital), UK. Patients were randomly assigned (1:1) using minimisation (gender and OSA severity) to receive TESLA or usual care (CPAP) for at least 3 months; sleep study analysis was provided without knowledge of the assignment arm. The primary outcome was change in AHI at 3-months. The primary outcome and safety were analysed in the intention-to-treat population. Data are reported as median (interquartile range), unless otherwise explained. This trial is registered at ClinicalTrials.gov, NCT03160456.

**Findings:**

Between 6 June 2018 and 7 February 2023, 56 participants were enrolled and randomly assigned (29 patients in the intervention group and 27 in the usual care group). Patients were followed up for a median of 3.0 months (IQR 3.0; 10.0). The groups were similar in terms of age (55.8 (48.2; 66.0) *vs* 59.3 (47.8; 64.4) years), gender (male:female, 19:10 *vs* 18:9) and BMI (28.7 (26.4; 31.9) *vs* 28.4 (24.4; 31.9) kg∗m^−2^). The unadjusted group difference in the ΔAHI was −11.5 (95% CI −20.7; −2.3) h^−1^ (p = 0.016). Adjusted for the baseline value, the difference was ΔAHI −7.0 (−15.7; 1.8) h^−1^ (p = 0.12), in favour of the intervention. Minor adverse events were found in one of the participants who developed mild headaches related to the intervention.

**Interpretation:**

Domiciliary TESLA can be used safely and effectively in OSA patients with poor adherence to CPAP, with favourable impact on sleepiness and sleep fragmentation. Despite pandemic-related limitations of the amended protocol this trial provides the evidence that TESLA improves clinically meaningful outcomes over the observed follow up period, and the transcutaneous approach is likely to offer an affordable alternative for responders to electrical stimulation in clinical practice.

**Funding:**

10.13039/501100000351British Lung Foundation, 10.13039/100011417United Kingdom Clinical Research Collaboration-registered King's Clinical Trials Unit at 10.13039/501100002102King's Health Partners.

**Research in context**

**Evidence before this study**

A meta-analysis (CRD42017074674) searched Medline/PubMed and the Cochrane Central Register of Controlled Trials (CENTRAL) to investigate the effects of hypoglossal nerve stimulation (HNS) on the apnoea-hypopnoea-index (AHI) and the Epworth Sleepiness Scale (ESS) in patients with OSA, published evidence was included up to May 2018. 41 clinical trials were identified, of which 20 interventional trials (n = 895) could be pooled in the meta-analysis, the majority using an invasive method of HNS. Middle-aged and overweight patients with severe OSA improved significantly in the AHI, with a larger effect size being observed using the invasive method when compared to the transcutaneous approach. The ESS improved by a clinically meaningful margin as well. However, there are no long-term follow up data of the transcutaneous approach in the domiciliary setting so far, and the recent European Respiratory Society (ERS) guidelines on non-CPAP therapy concluded that there was generally “very low quality of evidence” on HNS, including both the invasive and the transcutaneous approach. We sought to test how domiciliary transcutaneous electrical stimulation in patients with OSA who have very low CPAP adherence could improve the condition, as measured by the AHI, the oxygen desaturation index (ODI) and the Epworth Sleepiness Scale (ESS), over a three month period.

**Added value of this study**

This is the first study to show that domiciliary use of transcutaneous electrical stimulation of the submental area in OSA patients with very low CPAP adherence can improve sleep apnoea severity and associated sleepiness, with only minor adverse events related to skin discomfort and headache.

**Implications of all the available evidence**

The data of the current trial suggest that domiciliary transcutaneous electrical stimulation for patients with OSA who do not adhere to long-term CPAP therapy can be delivered safely and efficaciously over a period of three months, or longer.

## Introduction

Obstructive sleep apnoea (OSA) is common and affects up to 1 billion people worldwide,^1^ its prevalence has increased in recent decades due to the worldwide obesity pandemic.^2^^,^^3^ The standard treatment for OSA remains continuous positive airway pressure (CPAP) therapy^4^; however, mandibular advancement devices (MAD)^5^ and other non-CPAP therapies^6^ provide safe and effective alternative treatment options for selected sub-populations of patients with OSA. In the absence of meaningful weight change, OSA usually requires long-term treatment. A significant proportion of patients do not adhere to CPAP therapy,^7^^,^^8^ making the development of evidence-based second line therapy for OSA a priority.

Electrical stimulation to maintain upper airway dilator tone in patients with OSA has been described using transcutaneous (TESLA trial)^9^^,^^10^ and invasive (STAR trial)^11^ methods. Hypoglossal Nerve Stimulation (HNS) was approved for the treatment of OSA by the US Food and Drug Administration (FDA) in 2014, and further assessed by the UK National Institute for Health and Care Excellence (NICE) regarding safety and efficacy in 2017.^12^ However, it requires the implantation of a medical device with eligibility assessments (e.g., drug-induced sleep endoscopy, DISE), and adverse effects remain to be considered when compared to standard therapy, putting demand on healthcare resources. Transcutaneous electrical stimulation in sleep apnoea (TESLA) with the titration of the electrical current according to skin sensation,^13^ provides a similar and, potentially, more cost-efficient effect with little or no side effects.^9^^,^^10^^,^^14^^,^^15^

The aim of this trial was to test safety and efficacy of domiciliary TESLA in patients with OSA over a period of three months, with patients self-administering the stimulation using a standard transcutaneous electrical neurostimulator (TENS) machine. We hypothesised that nocturnal delivery of TESLA would reduce the severity of OSA, as measured by the apnoea-hypopnoea-index (AHI), and alleviate associated daytime symptoms; specifically sleepiness, as measured by the Epworth Sleepiness Scale (ESS), compared to usual care with CPAP.

## Methods

### Study design

This was a single-centre, open-label, randomised, controlled phase III trial, which was registered on ClinicalTrials.gov (NCT03160456), approved by the London-Dulwich, UK ethics committee (IRAS 217448) and the R&D department of Guy’s & St Thomas’ NHS Foundation Trust (GSTT), with the protocol published,^16^ and adhering to CONSORT reporting guidelines. Patients were screened for eligibility at the Sleep Disorders Centre at GSTT; informed written consent was obtained. The trial schedule included three months of intervention, with a sleep study at baseline and at follow-up on the allocated treatment, an outpatient visit at six weeks, and bi-weekly motivational phone calls (for more details please refer to the Online Supplement).

The trial started recruitment 18/06/2018 intending to conclude recruitment within 2.5 years. However, the trial was paused from March 2020 during the COVID pandemic, as GSTT transformed into a supra-regional centre for ventilated patients, with many staff being re-deployed to intensive care units. R&D only permitted COVID-related research at this time and, following loosening of the pandemic-related social distancing restrictions, an amendment to the trial protocol was submitted incorporating the latest infection prevention and control (IPC) guidance with COVID-safe procedures (home-based respiratory polygraphy, as opposed to the initially intended polysomnography). The amendment was approved by the ethics committee prior to opening recruitment again in March 2022, with the last participant exiting the trial on 08/02/2023 (sample size achieved).

### Participants

Patients with OSA (AHI 5–35/h) were included in this study if they failed to use CPAP therapy >4 h∗night^−1^, or who had withdrawn from standard therapy. Patients were required to have a body mass index (BMI) of 18.5–32.0 kg∗m^−2^ and upper airway anatomy without enlarged tonsils.

We excluded patients with exclusively postural OSA, defined as obstructive events in the supine and normal breathing in non-supine posture, exclusively Rapid-Eye-Movement (REM) sleep associated OSA, and patients with features of obesity hypoventilation syndrome (pCO_2_ >6.0 kPa, HCO3^−^ >28 mmol/L). Patients with enlarged tonsils (size 3–4), significant polyps and adenoids, neuromuscular disease, hypoglossal nerve palsy, clinically significant abnormal pulmonary function tests, severe pulmonary hypertension, valvular heart disease, heart failure (New York Heart Association, NYHA III–IV), acute myocardial infarction or significant cardiac arrhythmias, uncontrolled hypertension, active psychiatric disorder, co-existing non-respiratory sleep disorder, metal implants or cardiac pacemakers were excluded. In addition, patients had to be excluded if they had facial hair that precluded the correct placement of the hydrogel patch.

### Randomisation and masking

Following informed and written consent patients were assessed, including review of the pre-trial sleep study from their clinical services, and if eligible randomly assigned to one of two arms, A) intervention (domiciliary transcutaneous electrical stimulation, TESLA, while asleep), or B) usual care (ongoing Continuous Positive Airway Pressure, CPAP therapy) using tailored online randomisation software by the UK Clinical Research Collaboration-registered King’s Clinical Trials Unit at King’s Health Partners (Guy’s Hospital, London, UK; Q-049, 30/05/2018, V1.5.1). Groups were randomised 1:1 using minimisation by gender and OSA severity (AHI <15/h; AHI 15–35/h), as measured in the sleep study by the referring service. Scoring of the sleep studies was undertaken without knowledge of assigned trial arm.

### Procedures

Patients randomised into the intervention arm were assigned to 3 months of nocturnal bilateral transcutaneous submental electrical stimulation (TENS/EMS Premier Combo Plus®, Everyway Medical Instruments Co., Ltd., New Taipei City/Taiwan) using submental hydrogel patches at night, as previously described.^9^^,^^10^ The device is a widely available and programmable CE-marked device which stores usage data up to 30 days. The device does not provide telemetry read-out, however, the usage was checked during the face-to-face assessments by the research team; it was also reported to the study team during the telephone follow ups by the patient. A night-by-night assessment of usage is possible, and the provided average usage was calculated as total usage (in hours) divided by number of nights that the device should have been used for. Patients were educated about the device and encouraged to use it each night, titrating the current according to skin sensation to avoid awakening due to discomfort.^9^^,^^10^^,^^13^ The patients were also educated on placing the patches accordingly, and re-educated during the follow up sessions.^9^

Participants who were assigned to usual care were encouraged to continue to use their CPAP devices and received a repetition of the educational session, interface fitting and explanation of the benefits of treatment. Patients were followed up at 3-months (Online Supplement).

### Outcomes

The primary outcome parameter was the reduction in severity of OSA at 3-months compared to baseline, as defined by the apnoea-hypopnoea-index (AHI).

Secondary outcome parameters addressed sleepiness (ESS), the 4% oxygen desaturation index (ODI), compliance with treatment, as measured by the usage of transcutaneous electrical stimulation at night (total hours/per night); subjective comfort and acceptance were also assessed using a visual analogue scale (0–10 points), and adverse events were recorded.

A response to treatment was defined as a more than 50% reduction in the AHI from baseline and a total AHI <20/h, or a more than 25% reduction in the ODI, in line with previous trials.^10^^,^^11^

For the sleep study recording we used inpatient polysomnography until 2020 (Alice 6, Respironics, Murrysville, Pennsylvania, USA), and home-based respiratory polygraphy (Nox T3s, ResMed, Sydney, AUS) following the beginning of the COVID pandemic. All sleep studies were visually checked and manually scored by fully trained sleep technicians, before further reviewed by the research team (DR, JS). Criteria for the scoring of respiratory events were followed, as outlined in the American Academy of Sleep Medicine (AASM) manual for the scoring of sleep and associated events.^17^

### Statistical analysis

We performed a sample size analysis based on the previous trial by Strollo et al.^11^ using hypoglossal nerve stimulation in OSA. A minimum of 46 patients needed to enter this two-treatment parallel-design study to detect a clinically important difference of at least 12.3 units (h^−1^) and an assumed standard deviation of 11.8 unit (h^−1^) with a power of 90% and a significance level of 5%. Considering our experience with previous studies we needed to account for dropouts and loss-to-follow up (between 15 and 20%). However, we had a lower than expected dropout rate and concluded recruitment for the study when a total of 54 patient had completed the trial follow up period (decision by the trial steering committee; further information on the sample size calculation can be found in the Online Supplement).

Statistical analysis compared the change in the respective outcome parameters (primary outcome: ΔAHI; secondary outcomes: ΔESS, Δ4% ODI, compliance in hours usage/night) between intervention (active stimulation) and the control (usual care) group. An intention-to-treat analysis was performed. Continuous variables are presented as median and interquartile range (IQR), unless otherwise stated. To compare study groups, we used the Wilcoxon and paired t-test for continuous paired variables, and the χ^2^ test for categorical variables. Categorical data for the responder analysis (>50% improved AHI) was undertaken using the independent samples proportions test; Spearman correlations are reported for usage. Imputation of missing data of two patients in the intervention arm followed the a-priori defined no-change assumption. All analyses were performed using SPSS version 28.0.1.1 (IBM, NYC, NY/US). Data are reported as median (interquartile range), unless otherwise indicated. Differences were considered significant at p < 0.05.

### Role of the funding source

The funder of the study had no role in study design, data collection, data analysis, data interpretation, or writing of the report. The following authors have directly accessed and verified the data, and hold responsibility for the decision to submit for publication: DR, EN, MP, EIS, GK, JS.

## Results

**Fig. 1:**
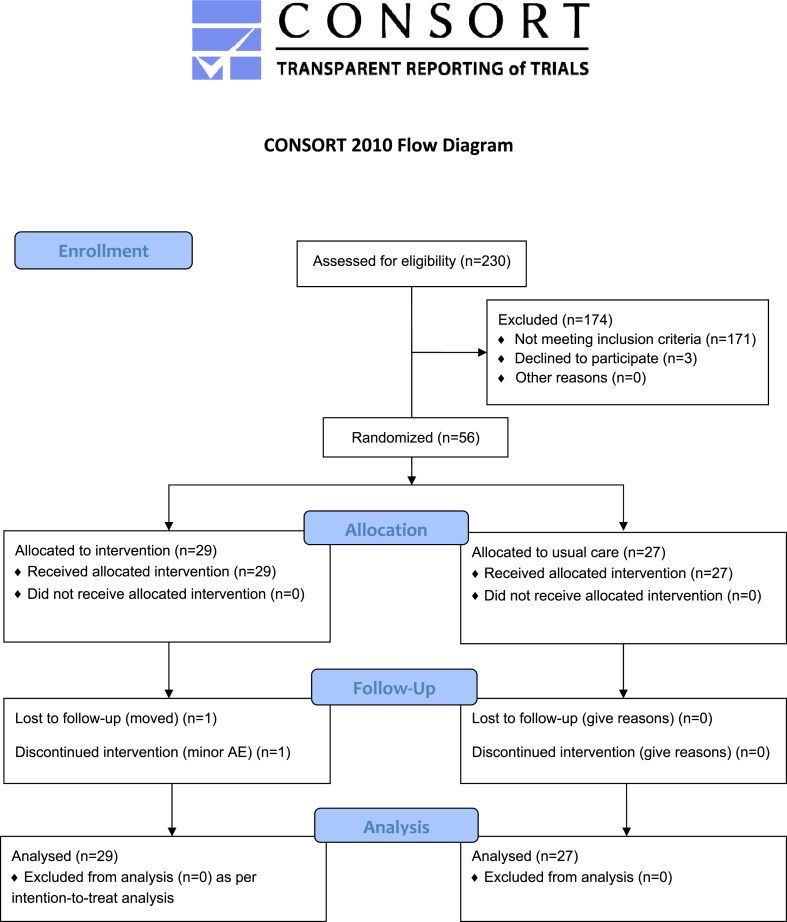
CONSORT diagram. Legend: Diagram including information on the screened cohort, patient allocation and final analysis. The patient who withdrew from the intervention experienced minor headaches. Analysis included all randomised patients in the intervention arm, as per '*intention-to-treat*' (n = 29). For two patients in the intervention arm the data for the follow up outcomes were imputed using the 'no-change' assumption, as specified a-priori. AE: adverse event.

A total cohort of 230 patients were screened between 6 June 2018 and 7 February 2023, 174 either did not meet the inclusion criteria or did not complete the consent form. The remaining 56 participants fulfilled all inclusion criteria, and completed the consent form. They were then randomised into two arms, intervention (n = 29) and usual care (n = 27). Two patients dropped out of the intervention arm following randomisation, one was lost to follow up after moving away, and one developed a minor headache and discontinued the intervention. The remaining 27 patients in the intervention group completed the trial (Fig. 1).

### Demographics

The intervention and the usual care group were well matched for age, gender, and body mass index (BMI). The groups were middle-aged, predominantly male, and within the overweight category. CPAP usage at randomisation was close to zero usage for both groups. At baseline assessment, patients had normal cardiovascular parameters, with no significant respiratory airway disease (Table 1).

**Table 1:** Group allocation characteristics.

Variable	Intervention (n = 29)	Usual care (n = 27)
Age (years)	55.8 (48.2; 66.0)	59.3 (47.8; 64.4)
Gender (m:f, n)	19:10	18:9
Ethnicity (white:black:Asian:mixed)	14:6:7:2	18:4:3:2
Height (m)	1.71 (1.65; 1.80)	1.74 (1.67; 1.83)
Weight (kg)	84.6 (78.5; 94.0)	83.0 (72.1; 96.0)
BMI (kg∗m^−2^)	28.7 (26.4; 31.9)	28.4 (24.4; 31.9)
Neck circumference (cm)	38 (36; 42)	39 (35; 42)
Waist (cm)	98 (93; 109)	98 (93; 110)
Hip (cm)	102 (96; 110)	102 (93; 105)
Waist:Hip ratio (a.u.)	0.99 (0.96; 1.03)	0.99 (0.93; 1.05)
Mallampati score (n for ‘1’/‘2’/‘3’)	10/13/6	8/14/5
Friedman score (n for ‘1’/‘2’/‘3’/‘4’)	20/6/2/1	18/8/1
CPAP usage (hours∗night^−1^)	0 (0; 0)	0 (0; 1)
Pulse (min^−1^)	79 (73; 85)	80 (72; 89)
Systolic blood pressure (mmHg)	133 (126; 140)	134 (120; 140)
Diastolic blood pressure (mmHg)	82 (75; 86)	79 (73; 87)
SpO_2_ (%)	98 (97; 99)	98 (96; 98)
FEV_1_ (L)	3.0 (2.5; 3.4)	3.0 (2.7; 3.8)
FVC (L)	3.4 (3.0; 4.1)	3.8 (3.3; 4.4)
FEV_1_/FVC (%)	85.6 (78.0; 89.8)	83.2 (77.2; 85.0)

Demographic of the allocated trial arm (intervention *vs* usual care) following the randomisation process. Patients were normotensive and had no significant respiratory condition.

n: number, m: male, f: female, BMI: body mass index, a.u.: arbitrary units, SpO_2_: oxygen saturation, FEV_1_: forced expiratory volume in 1 s, FVC: forced vital capacity.

Data presented as median (interquartile range) except for gender, ethnicity Mallampati and Friedman scores which are reported in numbers (n). Data are presented as median (interquartile range) unless indicated otherwise.

### Sleep study methodology

The COVID-19 pandemic put a halt to follow-up appointments and new recruitment into the trial from 03/2020, and caused a shift from inpatient polysomnography to home-based respiratory polygraphy. The first 23 participants completed both baseline and follow up inpatient polysomnography. However, the next 20 recruited participants had an initial polysomnography prior to pandemic lockdown, and following amendment of the protocol and re-opening of the trial in line with current infection prevention and control (IPC) guidance, a home-based polygraphy at follow up. Finally, the last 13 participants were recruited following permission of the R&D department in 03/2022 and received home-based polygraphy for both baseline and follow up study. With the randomization platform used (King’s Clinical Trials Unit) both trial arms (intervention and usual care) were similarly affected by the changes in the methodology of the sleep studies.

### Follow-up period

The follow up period was significantly impacted upon by the pandemic lockdown. The 20 participants who were consented and included in 03/2020 were kept ‘active’ in the study until they could be followed up safely. The median follow-up for the entire cohort was 3.0 (3.0; 10.0) months, with no significant differences between the intervention and usual care arms (3.0 (3.0; 5.5) *vs* 3.0 (3.0; 12.0) months; p = 0.37).

### Sleep study

#### Primary outcome

**Fig. 2:**
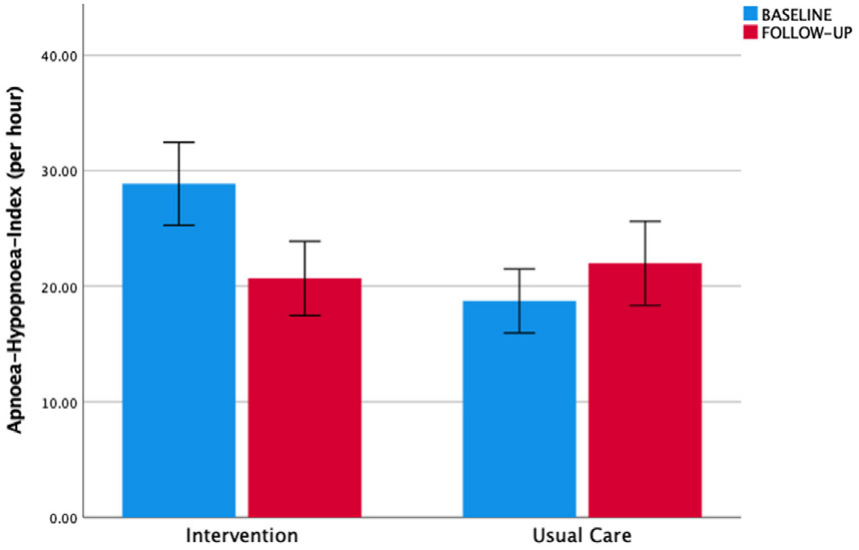
Apnoea-hypopnoea-index. Legend: Primary outcome (apnoea-hypopnoea-index, AHI) for intervention and usual care group at baseline (blue) and follow-up (red). There was a significant improvement in the intervention group (p = 0.006), but not the usual care group (p = 0.69). Bar plots represent mean values, and the T-error bars indicate the standard errors.

At baseline, patients in the intervention group had an AHI of 24.0 (13.5; 40.4) h^−1^ *vs* 14.4 (11.0; 21.0) h^−1^ in the usual care arm. At follow-up, the AHI had improved in the intervention (15.6 (9.2; 33.8) h^−1^; p = 0.006), but not in the usual care group (16.0 (8.6; 29.0) h^−1^; p = 0.69). The difference in the AHI in the intervention arm (ΔAHI −8.2 (95% CI −13.4; −3.0) h^−1^) *vs* usual care (ΔAHI + 3.3 (95% CI −4.7; 11.3) h^−1^) resulted in a mean group difference of −11.5 (95% CI −20.7; −2.3) h^−1^ for the AHI (unadjusted primary outcome; p = 0.016), and when adjusted for the baseline value it was −7.0 (−15.7; 1.8) h^−1^ (p = 0.12) (Fig. 2).

#### Secondary outcomes

**Fig. 3:**
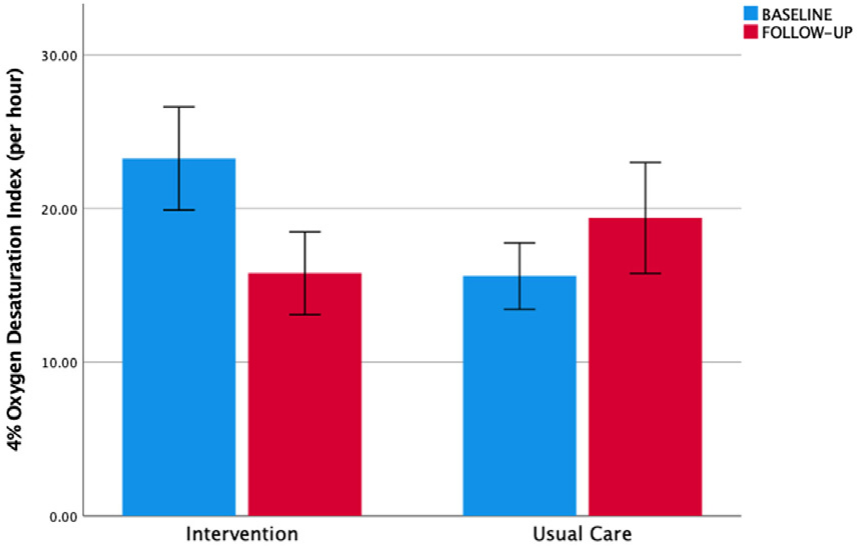
Oxygen desaturation index. Legend: Secondary outcome (4% oxygen desaturation index, ODI) for intervention and usual care group at baseline (blue) and follow-up (red) with an improvement in the intervention group (p < 0.004), but not the usual care group (p = 0.70). Bar plots represent mean values, and the T-error bars indicate the standard error.

The ODI in the intervention arm was 16.8 (8.6; 36.6) *vs* 10.2 (6.4; 23.1) h^−1^ in the usual care arm at baseline. The mean difference between the groups was −11.3 (95% CI −19.3; −3.2) h^−1^ (p = 0.007), and when adjusted for the baseline value −8.3 (−16.1; −0.6) h^−1^ in favour of the intervention (p = 0.036) (Fig. 3).

**Fig. 4:**
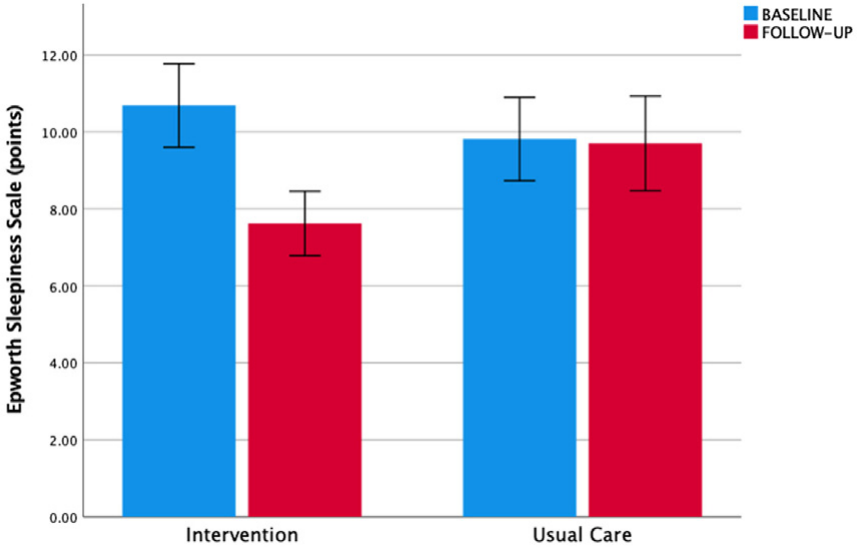
Epworth Sleepiness Scale. Legend: Secondary outcome (Epworth Sleepiness Scale, ESS) for intervention and usual care group at baseline (blue) and follow-up (red) with an improvement in the intervention group (p < 0.001). There was no significant change in the ESS in the usual care group (p = 0.86). Bar plots represent mean values, and the T-error bars indicate the standard error.

The ESS in the intervention group was initially 9.0 (5.5; 14.5) points, and 11.0 (6.0; 12.0) points in the usual care group. The mean group difference in the ESS at the end of the trial was −3.0 (95% CI −5.4; −0.5) points (p = 0.019), and when adjusted for the baseline value −2.6 (95% CI −4.9; −0.4) points (p = 0.020) in favour of the intervention (Table 2; Fig. 4).

**Table 2:** Between group change of outcome parameters.

	Intervention (n = 29)			Control group (n = 27)			Between-group change	
	BL	FU	Within-group change mean (SE)	BL	FU	Within-group change mean (SE)	Treatment effect (95% CI) p-value	Adjusted treatment effect^a^ (95% CI) p-value
AHI	28.9 (19.3)	20.7 (17.3)	−8.2 (2.6)	18.7 (14.4)	22.0 (18.9)	3.3 (3.9)	−11.5 (−20.7; −2.3) 0.016	−7.0 (−15.7; 1.8) 0.12
ODI	23.3 (18.1)	15.8 (14.5)	−7.5 (2.1)	15.6 (11.2)	19.4 (18.8)	3.8 (3.5)	−11.3 (−19.3; −3.2) 0.007	−8.3 (−16.1; −0.6) 0.036
ESS	10.7 (5.8)	7.6 (4.5)	−3.1 (0.9)	9.8 (5.6)	9.7 (6.4)	−0.1 (0.9)	−3.0 (−5.4; −0.5) 0.019	−2.6 (−4.9; −0.4) 0.020

The mean group differences for the intention to treat analysis between the intervention and the usual care group. Primary (AHI) and secondary outcomes (ODI, ESS) improved significantly in the intervention arm (pooled analysis). The primary outcome variable, the AHI, was significant for the unadjusted effect, but was not significant when adjusted for the baseline value (p = 0.12).

SE: standard error, 95% CI: confidence interval (lower and upper borders), AHI: apnoea-hypopnoea-index, ODI: 4% oxygen desaturation index, ESS: Epworth Sleepiness Scale, BL: baseline, FU: follow-up.

Variables for intervention and usual care arm are reported as mean (standard deviation), and for the within group change as mean (standard error of the mean).

a Treatment effect adjusted for baseline value.

Snoring in the intervention arm improved with the treatment by −26.9 (27.6) min (p < 0.001), and by −10.3 (11.4) percent of the total sleep time (p < 0.001). The improvement in snoring in the usual care group was dependent on whether CPAP was used at follow up, or not, and snoring improved by −3.2 (80.0) min (p = 0.84), and −5.7 (25.0) percent of the total sleep time at follow up (p = 0.25). However, the mean group difference of −23.6 (−8.0; 55.2) min (p = 0.14), and −4.6 (−5.7; 14.9) percent of the total sleep time (p = 0.37) did not reach significance (e-Table S2).

The five domains of the Functional Outcome of Sleep Questionnaire (FOSQ), vigilance, general productivity, social outcome, intimacy, activity, and the total score did not indicate any significant differences between the intervention and the usual care group during the follow up period (e-Table S3). Similarly, the five domains in the Euro-QoL-5D-5L (EQ-5D-5L; mobility, self-care, usual activities, pain/discomfort, anxiety/depression) as well as the associated visual analogue scale did not indicate any significant differences in the treatment effects between intervention and usual care arms (e-Table S4).

#### Adherence to treatment

At inclusion into the trial, CPAP usage was the same between the patients in the intervention arm and the usual care group (median of 0.0 (0.0; 0.0) *vs* 0.0 (0.0; 0.0) h). Applying a worst-case assumption of the loss-to-follow-up in the intervention arm, there was one patient who did not use the treatment, one patient who had been withdrawn, and one patient who was lost-to-follow-up (3/29; 10.3%). In the usual care group, there were more patients (16/27; 59.3%) who did not use CPAP therapy at all, despite encouragement (p < 0.001).

In the intervention arm, treatment was on average used for 4.0 (1.9; 4.9) days per week with a nightly use of 4.5 (2.8; 5.5) h. In contrast, patients in the usual care group did not use their treatment (CPAP), for 0.0 (0.0; 2.2) days per week (p < 0.001), with an average of 0.0 (0.0; 3.4) h per night of usage (p < 0.001).

In both arms, the nocturnal usage of treatment (hours/night) directly correlated with improved AHI (Spearman’s rho −0.494; p < 0.001), ODI (r = −0.533; p < 0.001), and ESS (r = −0.290; p = 0.030).

#### Responder analysis

In the responder analysis, 27.6% of the patients in the intervention group had a more than 50% reduction in the AHI (8/29), compared to 14.8% in the usual care group (4/27; p = 0.25). Using a different definition with a more than 25% reduction in the ODI 37.9% in the intervention arm classified as responders (11/29), and 22.2% in the usual care arm (6/27; p = 0.21). In contrast, 27.6% of patients in the intervention arm had an increase in the AHI at follow up (8/27) compared to 48.1% in the usual care group (13/27; p = 0.12). Considering the ODI, 24.1% had a higher index at follow-up with the intervention (7/29), while this was 44.4% in the usual care arm (12/27; p = 0.11).

Disease severity changed in the intervention group from 24.1% (7/29) with mild, 37.9% (11/29) with moderate, and 37.9% (11/29) with severe OSA at baseline to 3.4% (3/29) patients with no significant OSA, 34.5% (10/29) with mild, 27.6% (8/29) with moderate, and 27.6% (8/29) with severe OSA at follow up. In the usual care group, there were 55.6% (15/27) patients with mild OSA, 33.3% (9/27) with moderate, and 11.1% (3/27) with severe condition at baseline, and these proportions changed to 14.8% (4/27) of patients who had no OSA at follow up, 25.9% (7/27) with mild, 37.0% (10/27) with moderate, and 22.2% (6/27) with severe OSA at follow up. Respiratory control of the OSA in the usual care group was closely associated with uptake in CPAP adherence.

#### Adverse events

The trial period covered not only the initially anticipated 3-months, but due to the unexpected pandemic lockdown the final follow up period was substantially longer for the cohort of participants that were recruited into the trial during early 2020. This provided the opportunity to record adherence rates and adverse events over a sustained period while using a novel treatment in the domiciliary setting. The only observed adverse event (AE) was mild headaches in one participant. This participant was advised to stop the treatment, the headaches resolved, and no further AE were recorded during a span covering almost five years of the trial. In contrast, it should be mentioned that one participant reported a beneficial effect on headaches and migraine. Both participants were in the intervention group. Additional comments during the follow up calls were that the patches peeled off at night during hot summer weather (n = 3), and that they caused minor skin irritation (n = 3).

## Discussion

Domiciliary transcutaneous electrical stimulation for patients with OSA without significant comorbidities is feasible, safe, and reduces disease severity, as measured by the AHI, and sleepiness in patients who had very low adherence to CPAP therapy. This is of particular interest for those who have no significant upper airway obstruction (e.g., as measured by the Mallampati or Friedman scores). The group difference in the AHI, −11.5 (95% CI −20.7; −2.3) h^−1^, is sufficient to improve OSA severity, although evidence of an effect after adjustment for baseline value was less conclusive (p = 0.12). Domiciliary TESLA provides a feasible second line treatment for patients with OSA who have failed usual care, and who respond to the treatment over the first follow up period of about three months.

There were fewer non-responders in the intervention group, and fewer patients who deteriorated during the follow up period on the intervention when compared to the usual care group. More than a third of the intervention group (34.5%) had a positive response to the intervention, as defined by an improvement in the AHI ≥50%, and more than half when a decrease in the ODI of ≥25% was considered (55.2%). The remaining participants of the intervention group either did not change their AHI significantly compared to their baseline sleep study, or had a slight increase in the AHI at follow up (27.6%), while almost half of the usual care group deteriorated (48.1%).

Sleepiness, as measured by the Epworth Sleepiness Scale, also improved in the intervention arm by a clinically relevant margin of three points,^18^ while it did not improve in the usual care group. Improvements in the respiratory indices, but not in the symptom scores, were directly correlated to the duration on treatment in both trial arms.

Furthermore, snoring improved more consistently with usage of the assigned treatment in the intervention arm. Quality of life scores, as measured by the FOSQ and the EQ-5D-5L, did not indicate any differences between the treatments of usual care and intervention arm, which underlines the symptomatic non-inferiority of TESLA compared to ongoing CPAP therapy in this selected cohort of patients.

Non-CPAP therapy^6^^,^^19^ and endotyping of OSA^20^, ^21^, ^22^, ^23^ have attracted significant interest in recent years. This is an acknowledgement of limited CPAP compliance that impacts on a considerable proportion of patients with OSA.^7^^,^^8^ Hypoglossal nerve stimulation has been established in many public healthcare systems (e.g., US, Germany, UK) where it has been found to be cost-effective.^24^^,^^25^

Data from a randomised controlled trial stimulating the distal branch of the hypoglossal nerve in OSA, the STAR trial,^11^ have recently been supplemented by the publication of the THN3 trial, using targeted proximal hypoglossal nerve stimulation.^26^ Both methods describe a fall in the AHI, the STAR trial by −17.3 (95% CI −20.7; −14.9) h^−1^, and the THN3 trial by −14.4 (95% CI −18.0; −10.5) h^−1^. A different, non-randomised and single arm treatment trial using bilateral hypoglossal nerve stimulation, the BLAST OSA trial, reported a reduction in the AHI of −10.8 (95% CI −14.6; −7.0) h^−1^.^27^ The effect size of the implantable methods is therefore marginally better than the effect observed using a non-invasive approach with domiciliary TESLA.

Interestingly, targeted hypoglossal nerve stimulation of the proximal hypoglossal nerve, as used in the THN3 trial, activates not only protruding muscles exclusively, but leads to the contraction of a combination of lingual muscles and causes “*a stiffening rather than protruding*” of the tongue. It is also asynchronous, without the requirement to sense inspiration.^26^ This is consistent with the methodology used for domiciliary TESLA, which stimulates the entirety of the tongue muscles less specifically than a hypoglossal nerve implant, but in a similar asynchronous (non-triggered) manner, and with low levels of electrical current to avoid skin irritation and awakening. This concept was developed to impact on the compliance of the tongue with sustained neuromuscular tone, rather than producing visible contraction.^9^

However, surgical methods of hypoglossal nerve stimulation^11^^,^^26^^,^^27^ require multiple teams (e.g., Sleep Laboratory, ENT surgeon, Anaesthetist) with additional resources to usual care using drug-induced sleep endoscopy (DISE), endoscopy and operation theatre time, and follow up with post-interventional titration in the sleep laboratory. In contrast, using a transcutaneous approach with TESLA provides effective therapy for responders in a domiciliary setting, saving significant costs, resources and time.

Our group previously reported the results of the TESLA trial,^10^ a randomised, sham-controlled, double-blinded cross-over trial using transcutaneous electrical stimulation which used less restrictive screening parameters, and included patients with a higher BMI, larger neck circumference, and more severe OSA. We found a modest group effect of −4.1 (95% CI −8.9; 0.6) in the 4% oxygen desaturation index for all participants. However, we also reported on responders (17/36 patients) and found that the AHI improved by −9.1 (95% CI −16.2; −2.0) h^−1^ in this group. Following on from that study we designed the current trial to use inclusion and exclusion criteria based on the observation that less obese patients, with a smaller neck circumference and less severe OSA, are more likely to be responders to the transcutaneous method. Furthermore, we also had more female participants in the current trial (10/29 in the intervention arm) compared to the previous study (6/36), and female patients with OSA have typically slimmer neck lines than their male counterparts, explaining, in parts, the higher total effect size reported in the current trial.

More recently, a different investigator group reported in a randomised, controlled cross-over trial on intermittent and continuous transcutaneous electrical stimulation in OSA.^28^ They described a reduction in the AHI of −7.3 (95% CI −18.5; 3.9) h^−1^ for continuous, and −13.3 (95% CI −23.5; −3.1) h^−1^ for intermittent stimulation. Both effect sizes are within the range of our current findings, and highlight the importance to further refine electrical current specifications to optimise the method.

Furthermore, the comparison of the current trial’s data with pre-existing clinical trials, using invasive and non-invasive comparable methods, is consistent with findings in a recent meta-analysis describing the overall effect size.^15^ Electrical stimulation of the hypoglossal nerve is more efficacious with the invasive method, likely due to the closer and more targeted delivery of the current, while costs, ease-of-use, safety and non-invasive nature favour the transcutaneous approach. A trial comparing the invasive *vs* the transcutaneous approach could also answer whether both methods impact similarly on symptoms.

In the intervention arm, the side-effect spectrum was low and involved temporary headaches, skin irritation and loosening of the hydrogel patches, similar minor adverse effects to those described previously.^9^^,^^10^^,^^14^ Transcutaneous electrical neurostimulation has been used in various health conditions for decades, including neuromuscular pain and orthopaedic conditions; these devices are safe, effective, widely available with few contraindications and side effects and compare favourably in terms of costs and side effects against any implanted method.^11^^,^^25^^,^^27^ The availability and affordability make domiciliary TESLA a promising second line therapy in responders, particularly for those who have failed to adhere to first line therapy (e.g., CPAP, Mandibular Advancement Devices, MAD).

Electrical current specifications (e.g., frequency, current intensity, waveform, uni-*vs* bipolar, intermittent *vs* continuous) provides prospective options to further refine the method. A recent randomised controlled trial of the transcutaneous approach suggests that the application of intermittent electrical current can further improve efficacy,^28^ and future development of more tailored equipment and stimulation algorithms has the potential to provide further benefits of transcutaneous electrical stimulation.

The differences in the severity of OSA, as measured by the AHI, at baseline were randomly assigned and the primary outcome was the change in the AHI during the follow up period. While it improved with the intervention it did not do so in the usual care group, largely due to the low usage of CPAP in this cohort of patients. This is not unexpected and reflects the need to attend to these patients in the regular CPAP follow up of clinical services. Addressing treatment specific problems, investing in patient education, and the consideration of patient (and partner) health-beliefs need to be considered to develop a more complex intervention to improve adherence to CPAP therapy in future.

Our results confirm that it remains challenging to motivate patients to use CPAP therapy once they have failed to adhere to this first line therapy of OSA. However, it also highlights the willingness of patients to try emerging non-CPAP therapies that may provide therapeutic benefits (e.g., symptom control) beyond usual care.^29^ In this context, and considering all the evidence across the clinical outcomes measured in this trial, it is very likely that the intervention is effective for patients with OSA.

Secondly, patients for the trial were identified on the results of a single night’s sleep study from the referring sleep service. Patients were included in the trial if they matched the inclusion criteria (AHI 5−35 h^−1^). Subsequently, the baseline sleep study undertaken within the trial frequently showed different results of the AHI, leading to some patients being included who had a baseline AHI above 35 h^−1^. However, night-by-night variability of the AHI, with misclassification in the severity of OSA between 20–56% of the cases, has been well described elsewhere.^30^ Recording more than one night at baseline and follow up may resolve this issue in future.

We also understand that exclusion of a large portion of patients in clinical trials makes it difficult to generalise certain findings. However, we needed to acknowledge the experience from previous trials about potential responders to the treatment (e.g., patients with a lower BMI and lower neck circumference) which defined the selected inclusion and exclusion criteria, excluding patients with morbid obesity and more severe OSA. We also needed to consider many of the exclusion criteria in response to risk assessments required to address safety concerns of the respective regulatory bodies (e.g., hypoventilation syndrome with raised pCO_2_, seizures). Previous trials in the field had a ratio of screened-to-randomised patients of about 1:9^11^ or 1:10^10^; the current trial had a ratio of 1:4 (56: 230 patients). We therefore think that despite the list of exclusion criteria the included patient population was representative of the patients referred to our sleep centre–middle-aged, overweight or obese, and mobile.

Thirdly, thresholds to define responders do not necessarily indicate control of OSA but relative improvement. However, the criteria used were consistent with prior experience from physiological studies and clinical trials using transcutaneous electrical stimulation in OSA^9^^,^^10^^,^^28^ similar criteria were also used in clinical trials using invasive hypoglossal nerve stimulation.^11^^,^^26^^,^^27^

Inevitably, delays in the recruitment and randomisation caused by the pandemic-related pause contributed to longer than anticipated follow-up periods in both groups. This required a change in the protocol, which was approved by the NHS ethics and local R&D departments. However, the fact that patients were prepared to continue with the intervention throughout a prolonged lockdown period, and remained compliant with the treatment for about half a year (on average) underlines the acceptability of the intervention for domiciliary therapy.

In conclusion, domiciliary TESLA of the submental area is a feasible, safe and effective treatment for patients with OSA who do not tolerate CPAP in the long-term, and provides a potential second line alternative treatment for patients to improve pathophysiology and control symptoms.

## Contributors

Conception or design of the work: DR, MP, KH, JM, JS; Data acquisition: DR, MC, EN, RM, ES, JS; Data analysis: DR, EN, MP, KH, ES, GK, MIP, JM, JS; Interpretation of data for the work: DR, MC, RM, MP, KH, ES, YL, GK, MIP, JM, JS. All authors have drafted the work or revised it critically for important intellectual content; AND have provided their final approval of the version to be published; AND had had full access to the data and have agreed to be accountable for all aspects of the work in ensuring that questions related to the accuracy or integrity of any part of the work are appropriately investigated and resolved. The following authors have directly accessed and verified the data: DR, EN, MP, EIS, GK, JS.

## Data sharing statement

Data sharing will be considered according to ethical approval upon reasonable request from academic and clinical institutions via communication with the corresponding author (“with publication”), for post-hoc and meta-analysis with investigator support, and after approval of a written proposal (with a signed data access agreement).

## Declaration of interests

JS is the named inventor on a patent for an ‘apparatus for treatment of snoring and sleep apnoea’ (WO2016124739A1) that is owned by his employing organisations (Guy’s & St Thomas’ NHS Foundation Trust/King’s College London, UK). MIP holds a Consultancy contract with Phillips Respironics (CPAP devices). All other authors declare no competing interests.
