# Supplementary material for: Domiciliary transcutaneous electrical stimulation in patients with obstructive sleep apnoea and limited adherence to continuous positive airway pressure therapy: a single-centre, open-label, randomised, controlled phase III trial
Source: eClinicalMedicine. 2023 Aug 3;62:102112. doi: 10.1016/j.eclinm.2023.102112 (PMC10466238; doi:10.1016/j.eclinm.2023.102112)
Supplement: Online Supplement [file mmc1.pdf]

Online Supplement

Domiciliary transcutaneous electrical stimulation in patients with obstructive sleep apnoea and limited adherence to continuous positive airway pressure therapy: a single-centre, open-label, randomised, controlled phase 3 trial

Deeban Ratneswaran MBBS[1, 2], Michael Cheng MBBS[1,2], Ebrahim Nasser MBBS[1], Rajiv Madula MBBS[2], Martino Pengo PhD [2,3], Kath Hope ALCM TD[2,4], Esther I Schwarz PhD[2,5], Yuanming Luo PhD[1,6], Georgios Kaltsakas PhD[1,2], Michael I Polkey PhD[7], John Moxham MD[1], Joerg Steier PhD[1,2]

[1] King's College London, Faculty of Life Sciences and Medicine, Centre for Human & Applied Physiological Sciences, London, UK

[2] Lane Fox Unit / Sleep Disorders Centre, Guy's & St Thomas' NHS Foundation Trust, London, UK

[3] Istituto Auxologico Italiano IRCCS, University of Milan, Milan, Italy

[4] Hope2Sleep Patient Charity, Hull, UK

[5] Department of Pulmonology, University Hospital Zurich and University of Zurich, Zurich, Switzerland

[6] State Key Laboratory of Respiratory Disease, Guangzhou Medical University, Guangzhou, China

[7] Royal Brompton & Harefield Campus, Guy's & St Thomas' NHS Foundation Trust, London, UK

Table of Content

Page 1	**Title Page** (online supplement)
Page 2	**Table of Content**
Page 3	**e-Methods** (Details of Assessments)
Page 3	*Baseline Assessment (visit 1)*
Page 3	*Bi-weekly Telephone Contacts*
Page 3	*Follow up at 6-weeks (visit 2)*
Page 4	*Follow up at 12-weeks (visit 3)*
Page 4	**Sample Size**
Page 4	**Statistical Considerations**
Page 5	**e-Results**
Page 5	**Summary of Missing values** (Figure e-1)
Page 5	**Classification of OSA** (Figure e-2)
Page 6	**'Per-Protocol' Analysis of Outcomes** (Table e-1)
Page 7	**Snoring Data** (Table e-2)
Page 7	**Functional Outcomes of Sleep Questionnaire Data** (Table e-3)
Page 8	**Euro-QoL-5D-5L** (Table e-4)
Page 9	**Reference List**
Page 10	**Ethics Committee Approval**
Page 14	**Health Research Authority Approval**

e-Methods

Further details on the individual assessments are provided below.

Baseline Assessments (Visit 1)

The initial assessment included an overnight sleep study (polysomnography, PSG prior to the COVID pandemic / home-based respiratory polygraphy during and following the pandemic). Patients were assessed using the following parameters:

- Demographics, including age, gender, ethnicity, height, weight, BMI, waist, hip, waist:hip ratio,
- Upper airway inspection, including the Mallampati and Friedman scores,
- Pulse oximetry spot check, and blood pressure measurements,
- Sleep study parameters, as measured by inpatient polysomnography until 2020 (Alice 6, Respironics, Murrysville, Pennsylvania, USA), and home-based respiratory polygraphy (Nox T3s, ResMed, Sydney, AUS) following the beginning of the COVID pandemic.
- Symptoms, as measured by the Epworth Sleepiness Scale (ESS), and qualitative overnight report.

These visits took place with face-to-face appointments until the first pandemic lockdown (03/2020). Following R&D approval of the pandemic-related Infection Prevention Control (IPS) amendment the inpatient polysomnography was converted into a home-based respiratory polygraphy (from 03/2022 onwards).

Bi-weekly Telephone Contacts

All patients in both trial arms received bi-weekly phone calls to encourage usage of the device or CPAP therapy and discuss problems with the treatment. Patients were asked about comfort and adverse events / serious adverse events using a standardized interview pro-forma (ESS). During the pandemic-related pause several patients remained over prolonged periods in the follow up for the trial, encouraged to participate they were re-assessed when R&D permitted the final home-based sleep study.

Follow-up at 6-weeks (Visit 2)

At six weeks, patients were followed-up in the outpatient clinic at the Lane Fox Unit / Sleep Disorders Centre prior to the beginning of the pandemic, and remotely following the outbreak of COVID (03/2020). The following assessments were made (same variables as during baseline visit):

a) Demographics,

c) Symptoms,

d) Usage and compliance with medical device and treatment,

e) Adverse events

At this stage, the patients were educated on the device/CPAP again and encouraged to continue with usage.

Follow-up at 12-weeks / End of Trial

At the 12-week follow up the patients were invited to attend the Lane Fox Unit / Sleep Disorders Centre for a repeat overnight polysomnography prior to the COVID pandemic, and for a home-based respiratory polygraphy following the pandemic-related amendments in the protocol. The assessment repeated the baseline measurements and participants were encouraged to use the respective therapy, corresponding to their assigned intervention arm. Patients were then given a debriefing by the study team and individual study reports could be requested at this stage.

Sample Size Calculation

We performed a sample size analysis based on the previous trial by Strollo et al (11) using hypoglossal nerve stimulation in obstructive sleep apnoea. A total of 46 patients needed to enter this two-treatment parallel-design study. This sample size achieved 90% power to detect a clinically important difference in the apnoea-hypopnoea index of at least 12.3 hour^{-1} between the null hypothesis that both group means are 32.0 (h^{-1}) and the alternative hypothesis that the mean of the trial treatment group is 19.7 (h^{-1}) with estimated group standard deviations of 11.8 (h^{-1}), and with a significance level of 5%. These calculations were based on 1,000 Monte Carlo samples from

normal distributions and using a two-sided Mann-Whitney Test. To adjust for the unknown distribution of the primary outcome and based on the lower bound for the asymptotic relative efficiency of the Mann-Whitney U test, we have increased the sample size by a further 15% to 54. Considering our experience with previous studies over this time period we initially accounted for dropouts and loss-to-follow up of between 15-20% and proposed to study up to a total of 68 patients for this study. However, despite the R&D mandated pause to the study during the COVID pandemic we had a lower than expected dropout rate and concluded recruitment for the study when a total of 56 patient had entered the trial, with 54 completing the trial follow up period (joint decision by the trial steering committee).

Statistical Considerations

Statistical analysis compared the change in the respective outcome parameters (primary outcome: ΔAHI; secondary outcome: ΔESS, Δ4%ODI, compliance in hours usage/night) between intervention (active stimulation) and the control (usual care) group. Full statistical description was provided and for each of the variables analysed, univariate descriptive statistics was presented with an overview of the data. Continuous variables were presented as median and interquartile range (IQR), unless otherwise stated. For categorical variables, frequency counts and percentages were presented as summary statistics for the subgroups of interest. To compare study groups, we used the Wilcoxon and paired t-test for continuous paired variables, and the χ^2 test for categorical variables. The primary outcome parameter (AHI) was analysed using the Mann-Whitney test between treatment group (active stimulation, intervention) and control group (usual care). Similar analyses were conducted for secondary outcomes. Categorical data for the responder analysis (>50% improved AHI) was undertaken using the independent samples proportions test. The trial followed-up each patient for at least 12 weeks, and one patient was inevitably lost to follow-up due to moving away, while another one discontinued the intervention (both intervention arm). Sample size estimation had assumed that 15-20% of patients would not provide end of study information that could be evaluated. If this rate had been observed, data for many patients would have only partially been recorded. It was therefore prospectively agreed that for patients who withdrew or dropped out before the end of the study, the "no change assumption" was used to impute the missing subsequent values; at the end of the trial it was necessary to impute the data for the two patients that did not complete the protocol. This could have introduced a bias if the main reason for drop-out was deterioration. To examine this possibility, sensitivity analysis was performed to assess the primary efficacy outcome, however, the use of different imputation methods including "best-case scenario", and "worst- case scenario" possible scores for the missing data, as well as Markov Chain Monte Carlo (MCMC) Multiple Imputation and linear regression methods were not applied due to the small number of participants with missing data. Furthermore, we present the 'intention-to-treat' analysis including all randomised patients (Table 2), as well as the 'per-protocol' analysis with only those participants who completed the follow up (e-Table 1). All analyses were performed using SPSS version 28.0.1.1 (IBM, NYC, NY/US). For group differences the 95% confidence intervals (CI) are reported. Differences were considered significant at $p<0.05$.

e-Results

The COVID-19 pandemic had an impact on the diagnostical pathway of the initial protocol that was used in the trial. Due to infection prevention and control (IPC) measures by the hospital the protocol needed to be amended from inpatient polysomnographies at baseline and follow-up. Following approval by the Trust IPC and R&D department, and health related authorities (ethics committee) we introduced home testing with respiratory polygraphies.

This had an impact on the diagnostics used in the trial. Out of the 56 recruited subjects, 25 had polysomnographies at baseline and follow up (completed before 03/2020), 20 patients had an initial polysomnography at baseline and required a home-based polygraphy at follow up (recruited prior to 03/2020, but not completed), and 11 participants had home-based studies at baseline and follow up (recruited following reopening of the trial in 03/2022).

We compared the ratios of different sleep studies used between the intervention and the usual care arm. However, we found no significant differences in the diagnostics used between the groups, likely due to the 1:1 randomisation which kept the time effect (pause: 03/2020 – 03/2022) similar in both assigned trial arms.

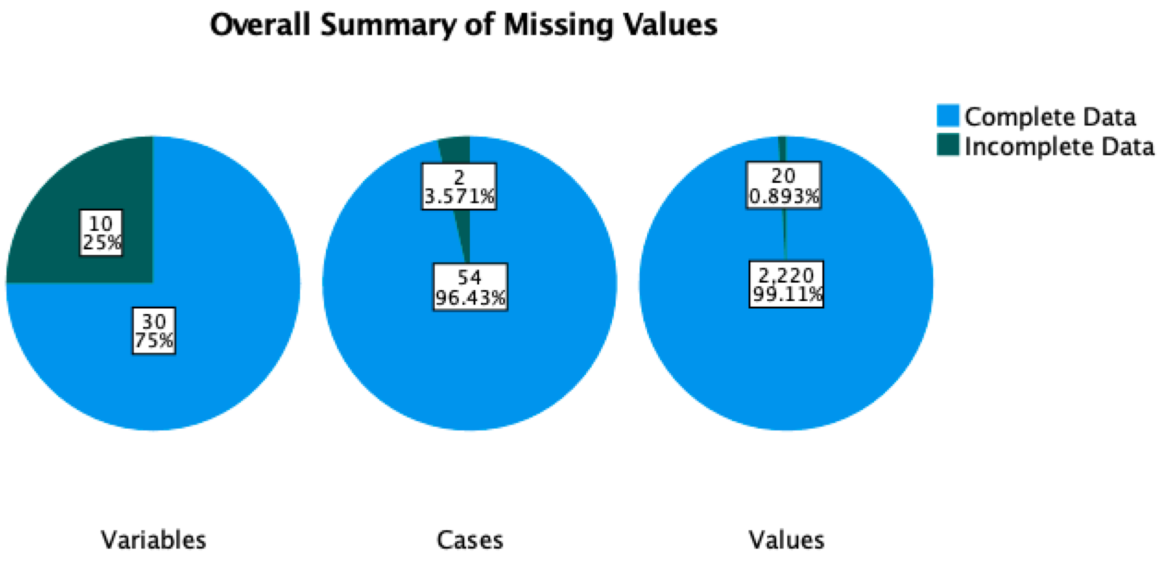

e-Figure 1: The missing values analysis for the intention to treat analysis revealed that 10 variables were affected by the two patients who did not complete the protocol in the intervention arm (3.6% of the cases), missing a total of 0.89% of all values.

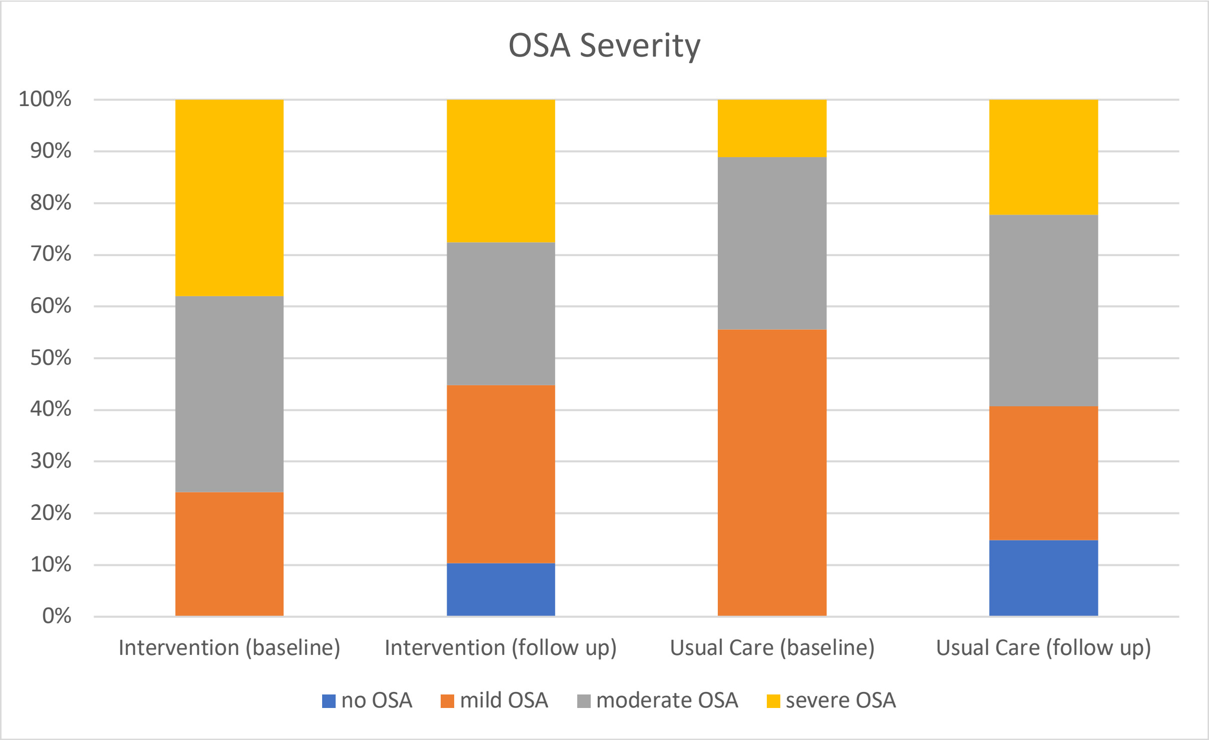

e-Figure 2: OSA classification with mild, moderate and severe OSA, according to assigned trial arm (intervention vs usual care) at baseline and follow up. At follow up, there are some patients (blue) in both groups who are perfectly controlled with no significant OSA on the treatment. While this indicates effectiveness in these responders in the intervention arm, it was indicative of the uptake of the CPAP therapy in the usual care group.

‘Per Protocol’ Analysis

Following analysis of the missing values (Figure e1) and to further explore whether there were any significant differences caused by the non-completion of two participants in the intervention arm we undertook a ‘per-protocol’ analysis and compared the results against the ‘intention-to-treat’ analysis. However, there were modest and non-significant changes in the outcomes (Table e1).

	Intervention (n=27)			Control group (n=27)			Between-group change	
	**BL**	**FU**	**Within-group change mean (SE)**	**BL**	**FU**	**Within-group change mean (SE)**	**Treatment effect (95%CI) p-value**	**Adjusted treatment effect* (95%CI) p-value**
AHI	30.0 (19.6)	21.1 (17.8)	-8.8 (2.7)	18.7 (14.3)	22.0 (18.9)	3.3 (3.9)	-12.1 (-21.6; -2.6) p=0.014	-7.1 (-16.2; 2.0) p=0.124
ODI	24.1 (18.4)	16.0 (15.0)	-8.0 (2.2)	15.6 (11.2)	19.4 (18.8)	3.8 (3.5)	-11.8 (-20.1; -3.5) p=0.006	-8.6 (-16.7; -0.5) p=0.038
ESS	10.8 (6.1)	7.5 (4.7)	-3.3 (0.9)	9.8 (5.6)	9.7 (6.4)	-0.1 (0.9)	-3.2 (-5.7; -0.7) p=0.014	-2.8 (-5.1; -0.6) p=0.016

e-Table 1: Primary (Δ apnoea-hypopnoea index, AHI) and secondary (4% oxygen desaturation index, ODI, and Epworth Sleepiness Scale, ESS) outcome parameters, as ‘per-protocol’ analysis of those patients that completed the entire intervention protocol. This analysis provided included ‘complete cases’ with ‘observed data’, no data imputation was required. The results are similar when compared to the ‘intention-to-treat’ analysis, with modest and non-significant changes in the decimals. SE=standard error. BL=baseline. FU=follow-up. * adjusted for baseline value. 95% CI=95% Confidence Interval.

Snoring

Patients had the tendency to snore less when established on the treatment at follow up. In both groups, the snoring indices improved slightly with treatment compared to the baseline sleep study, but there was no significant difference between the groups. Particularly the results in the usual care group varied widely, depending on whether patients did use CPAP therapy at follow up or not, while the treatment effect in the intervention group was more consistently unidirectional (e-Table 2).

Variable	Intervention (n=29)	Usual Care (n=27)	Mean Difference	SE Difference	95% CI Difference	p-value (two-sided)
**Snoring (mins)**	-26·9 (27·6)	-3·2 (80·0)	-23·6	15·8	-8·0; 55·2	0·139
**Snoring (%TST)**	-10·3 (11·4)	-5·7 (25·0)	-4·6	5·1	-5·7; 14·9	0·371

e-Table 2: The change in recorded snoring (mean, standard deviation), as measured in minutes overnight or as percentage of the total sleep time (%TST), improved slightly in both trial arms, particularly when the treatment was used at follow up. However, there was no significant difference between the intervention or usual care arm. SE=standard error. 95%CI=Confidence Interval.

Functional Outcome of Sleep Questionnaire (FOSQ)

We assessed quality of life using the long version (30 items) of the FOSQ at baseline and follow up.[1] The differences in the domains (vigilance, general productivity, social outcome, intimacy, activity), and in the total scores during the trial period did not differ significantly between the intervention and usual care group (e-Table 3).

FOSQ Variable	Intervention (n=29)	Usual Care (n=27)	Mean Difference	SE Difference	95% CI Difference	p-value (two-sided)
**Vigilance**	0·000 (0·845)	0·185 (0·396)	0·185	0·179	-0·173; 0·543	0·304
**General Productivity**	0·207 (0·675)	0.074 (0·550)	-0·133	0·165	-0·464; 0·198	0·425
**Social Outcome**	0·035 (0·499)	0·148 (0·456)	0·114	0·128	-0·143; 0·370	0·379
**Intimacy**	-0·035 (0·421)	0·037 (0·338)	0·072	0·102	-0·134; 0·267	0·488
**Activity**	0·069 (0·530)	0·148 (0·602)	0·079	0·151	-0·224; 0·382	0·603
**Total score**	0·276 (2·202)	-0·012 (1·526)	0·317	0·510	-0·706; 1·328	0·537

e-Table 3: The change in the five domains (mean, standard deviation) of the Functional Outcome of Sleep Questionnaire (FOSQ) and the total score did not indicate any significant differences between the intervention and the usual care group. SE=standard error. 95%CI=Confidence Interval.

EQ-5D-5L

In an attempt to further assess the quality of life we measured the European Quality of Life (Euro-QoL) questionnaire, measuring five domains with five levels (EQ-5D-5L).[2] The five domains included mobility, self-care, usual activities, pain/discomfort, anxiety/depression. All domains can be scored from 1-5, with lower indices indicating better health. The EQ-5D-5L also contains a visual analogue scale (VAS 0-100) with higher scores indicating better health. We compared the change in outcomes between the intervention and the usual care groups during the trial period, but there was no significant difference (e-Table 4).

FOSQ Variable	Intervention (n=29)	Usual Care (n=27)	Mean Difference	SE Difference	95% CI Difference	p-value (two-sided)
**Mobility**	0·00 (0·38)	-0·07 (0·27)	-0·074	0·088	-0·251; 0·102	0·404
**Self-care**	-0·03 (0·42)	-0·07 (0·27)	-0·040	0·087	-0·230; 0·151	0·679
**Usual Activities**	-0·14 (0·64)	-0·07 (0·38)	0·064	0·142	-0·222; 0·349	0·656
**Pain/ discomfort**	0·17 (0·54)	-0·04 (0·17)	-0·209	0·167	-0·545; 0·126	0·216
**Anxiety/ depression**	-0·03 (0·63)	-0·30 (0·71)	-0·262	0·188	-0·638; 0·114	0·169
**VAS**	0·83 (10·31)	2·44 (18·28)	1·617	3·930	-6·262; 9·496	0·682

e-Table 4: The change in the five domains (mean, standard deviation) of the EQ5D-5L (1-5), and the visual analogue scale (VAS, 0-100). There were no significant differences between the intervention and the usual care group. SE=standard error. 95%CI=Confidence Interval.

References

1) Weaver TE, Laizner AM, Evans LK, Maislin G, Chugh DK, Lyon K, Smith PL, Schwartz AR, Redline S, Pack AI, Dinges DF. An instrument to measure functional status outcomes for disorders of excessive sleepiness. Sleep. 1997 Oct;20(10):835-43. PMID: 9415942.
2) EuroQol Research Foundation. EQ-5D-5L User Guide, 2019. Available from: **https://euroqol.org/publications/user-guides**. accessed 20/06/2023

NHS
Health Research Authority

London - London Bridge Research Ethics Committee
Skipton House
80 London Road
London
SE1 6LH

Telephone: 020 7104 8222
Fax:020 7104 8222

<u>Please note</u>: This is the favourable opinion of the REC only and does not allow you to start your study at NHS sites in England until you receive HRA Approval

05 June 2018

Dr Joerg Steier
Consultant Physician and Reader in Respiratory and Sleep Medicine
Guy's and St Thomas' NHS Foundation Trust
Lane Fox Unit / Sleep Disorders Centre
Guy's and St Thomas' NHS Foundation Trust
Westminster Bridge Road, London
SE1 7EH

Dear Dr Steier

**Study title:**	**Randomised controlled trial of domiciliary transcutaneous electrical stimulation in obstructive sleep apnoea: TESLA-home**
**REC reference:**	**18/LO/0638**
**IRAS project ID:**	**217448**

Thank you for your letter responding to the Committee's request for further information on the above research and submitting revised documentation.

The further information has been considered on behalf of the Committee by the Chair.
We plan to publish your research summary wording for the above study on the HRA website, together with your contact details. Publication will be no earlier than three months from the date of this opinion letter. Should you wish to provide a substitute contact point, require further information, or wish to make a request to postpone publication, please contact hra.studyregistration@nhs.net outlining the reasons for your request.

Confirmation of ethical opinion

On behalf of the Committee, I am pleased to confirm a favourable ethical opinion for the above research on the basis described in the application form, protocol and supporting documentation, subject to the conditions specified below.

Management permission must be obtained from each host organisation prior to the start of the study at the site concerned.

Management permission should be sought from all NHS organisations involved in the study in accordance with NHS research governance arrangements. Each NHS organisation must confirm through the signing of agreements and/or other documents that it has given permission for the research to proceed (except where explicitly specified otherwise).

Guidance on applying for HRA and HCRW Approval (England and Wales)/ NHS permission for research is available in the Integrated Research Application System, at www.hra.nhs.uk or at http://www.rdforum.nhs.uk.

Where a NHS organisation's role in the study is limited to identifying and referring potential participants to research sites ("participant identification centre"), guidance should be sought from the R&D office on the information it requires to give permission for this activity.

For non-NHS sites, site management permission should be obtained in accordance with the procedures of the relevant host organisation.

Sponsors are not required to notify the Committee of management permissions from host organisations

Registration of Clinical Trials

All clinical trials (defined as the first four categories on the IRAS filter page) must be registered on a publically accessible database within 6 weeks of recruitment of the first participant (for medical device studies, within the timeline determined by the current registration and publication trees).

There is no requirement to separately notify the REC but you should do so at the earliest opportunity e.g. when submitting an amendment. We will audit the registration details as part of the annual progress reporting process.

To ensure transparency in research, we strongly recommend that all research is registered but for non-clinical trials this is not currently mandatory.

If a sponsor wishes to request a deferral for study registration within the required timeframe, they should contact hra.studyregistration@nhs.net. The expectation is that all clinical trials will be registered, however, in exceptional circumstances non registration may be permissible with prior agreement from the HRA. Guidance on where to register is provided on the HRA website.

It is the responsibility of the sponsor to ensure that all the conditions are complied with before the start of the study or its initiation at a particular site (as applicable).

Ethical review of research sites

NHS sites

The favourable opinion applies to all NHS sites taking part in the study, subject to management permission being obtained from the NHS/HSC R&D office prior to the start of the study (see "Conditions of the favourable opinion" below).

Approved documents

The final list of documents reviewed and approved by the Committee is as follows:

Document	*Version*	*Date*
Copies of advertisement materials for research participants [Webpage Advertisement Text - V1.0 25-07-2017]	1.0	25 July 2017
Covering letter on headed paper [Cover Letter]	1.0	09 August 2017
Covering letter on headed paper		10 May 2018
GP/consultant information sheets or letters [GP Consultant Information Sheet or Letter]	1.0	09 August 2017
Instructions for use of medical device [User manual device]	Doc ZS-P-08-D	
Interview schedules or topic guides for participants [Interview Schedules]	1.0	09 August 2017
IRAS Application Form [IRAS_Form_06032018]		06 March 2018
Letter from statistician [Statistician's Input]	1.0	09 August 2017
Letters of invitation to participant	1.1	10 May 2018
MHRA Notice of No Objection Letter (Medical Devices) and relevant correspondence [MHRA >>letter of no objection/email<<]	MHRA ref: E/2017/0126	10 February 2017
Other [Visual Analogue Score]		
Participant consent form	1.1	10 May 2018
Participant information sheet (PIS)	1.1	10 May 2018
Research protocol or project proposal	1.1	14 May 2018
Summary CV for Chief Investigator (CI) [CV Chief Investigator]	1.0	16 May 2017
Summary, synopsis or diagram (flowchart) of protocol in non technical language [Summary Non-Expert]	1.0	09 August 2017
Validated questionnaire [Epworth Sleepiness Scale]	1.0	04 July 2017
Validated questionnaire [EQ-5D-5L]		
Validated questionnaire [Stanford Sleepiness Scale]	1.0	04 July 2017
Validated questionnaire [Hospital Anxiety and Depression Scale]	1.0	04 July 2017
Validated questionnaire [Functional Outcomes Sleep Questionnaire]	1.0	04 July 2017

Statement of compliance

The Committee is constituted in accordance with the Governance Arrangements for Research Ethics Committees and complies fully with the Standard Operating Procedures for Research Ethics Committees in the UK.

After ethical review

<u>Reporting requirements</u>

The attached document *"After ethical review – guidance for researchers"* gives detailed guidance on reporting requirements for studies with a favourable opinion, including:

- Notifying substantial amendments
- Adding new sites and investigators
- Notification of serious breaches of the protocol
- Progress and safety reports
- Notifying the end of the study

The HRA website also provides guidance on these topics, which is updated in the light of changes in reporting requirements or procedures.

User Feedback

The Health Research Authority is continually striving to provide a high quality service to all applicants and sponsors. You are invited to give your view of the service you have received and the application procedure. If you wish to make your views known please use the feedback form available on the HRA website:
http://www.hra.nhs.uk/about-the-hra/governance/quality-assurance/

HRA Training

We are pleased to welcome researchers and R&D staff at our training days – see details at
http://www.hra.nhs.uk/hra-training/

**18/LO/0638**	**Please quote this number on all correspondence**

With the Committee's best wishes for the success of this project.

Yours sincerely
PP:

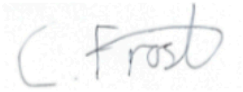

Ms Jane Smith
Chair

Email:nrescommittee.london-londonbridge@nhs.net

Dr Joerg Steier
Consultant Physician and Reader in Respiratory and Sleep Medicine
Guy's and St Thomas' NHS Foundation Trust
Lane Fox Unit / Sleep Disorders Centre
Guy’s and St Thomas’ NHS Foundation Trust
Westminster Bridge Road, London
SE1 7EH

Email: hra.approval@nhs.net
Research-permissions@wales.nhs.uk

06 June 2018

Dear Dr Steier

HRA and Health and Care Research Wales (HCRW) Approval Letter

**Study title:**	**Randomised controlled trial of domiciliary transcutaneous electrical stimulation in obstructive sleep apnoea: TESLA-home**
**IRAS project ID:**	**217448**
**REC reference:**	**18/LO/0638**
**Sponsor**	**Guy's and St Thomas' NHS Foundation Trust**

I am pleased to confirm that HRA and Health and Care Research Wales (HCRW) Approval has been given for the above referenced study, on the basis described in the application form, protocol, supporting documentation and any clarifications received. You should not expect to receive anything further relating to this application.

How should I continue to work with participating NHS organisations in England and Wales?
You should now provide a copy of this letter to all participating NHS organisations in England and Wales, as well as any documentation that has been updated as a result of the assessment.

Following the arranging of capacity and capability, participating NHS organisations in England and Wales that are participating as Research Sites should **formally confirm** their capacity and capability to undertake the study. How this will be confirmed is detailed in the “*summary of assessment*” section towards the end of this letter. You should then work with each organisation that has confirmed capacity and capability and provide clear instructions when research activities can commence.

Participating NHS organisations in England and Wales that are participating as PIC sites **will not** be required to formally confirm capacity and capability before you may commence research activity at site. As such, you may commence the research at each organisation 35 days following sponsor provision to the site of the local information pack, so long as:

- You have contacted participating NHS organisations (see below for details)
- The NHS organisation has not provided a reason as to why they cannot participate
- The NHS organisation has not requested additional time to confirm.

You may start the research prior to the above deadline if the site positively confirms that the research may proceed.

If not already done so, you should now provide the local information pack for your study to your participating NHS organisations. A current list of R&D contacts is accessible at the NHS RD Forum website and these contacts MUST be used for this purpose. After entering your IRAS ID you will be able to access a password protected document (password: **Whale33**). The password is updated on a monthly basis so please obtain the relevant contact information as soon as possible; please do not hesitate to contact me should you encounter any issues.

Commencing research activities at any NHS organisation before providing them with the full local information pack and allowing them the agreed duration to opt-out, or to request additional time (unless you have received from their R&D department notification that you may commence), is a breach of the terms of HRA and HCRW Approval. Further information is provided in the "*summary of assessment*" section towards the end of this document.

It is important that you involve both the research management function (e.g. R&D office) supporting each organisation and the local research team (where there is one) in setting up your study. Contact details of the research management function for each organisation can be accessed here.

How should I work with participating NHS/HSC organisations in Northern Ireland and Scotland?
HRA and HCRW Approval does not apply to NHS/HSC organisations within the devolved administrations of Northern Ireland and Scotland.

If you indicated in your IRAS form that you do have participating organisations in either of these devolved administrations, the final document set and the study wide governance report (including this letter) has been sent to the coordinating centre of each participating nation. You should work with the relevant national coordinating functions to ensure any nation specific checks are complete, and with each site so that they are able to give management permission for the study to begin.

Please see IRAS Help for information on working with NHS/HSC organisations in Northern Ireland and Scotland.

How should I work with participating non-NHS organisations?
HRA and HCRW Approval does not apply to non-NHS organisations. You should work with your non-NHS organisations to obtain local agreement in accordance with their procedures.

What are my notification responsibilities during the study?
The document "*After Ethical Review – guidance for sponsors and investigators*", issued with your REC favourable opinion, gives detailed guidance on reporting expectations for studies, including:

- Registration of research
- Notifying amendments
- Notifying the end of the study

The HRA website also provides guidance on these topics, and is updated in the light of changes in reporting expectations or procedures.

I am a participating NHS organisation in England or Wales. What should I do once I receive this letter?
You should work with the applicant and sponsor to complete any outstanding arrangements so you are able to confirm capacity and capability in line with the information provided in this letter.

The sponsor contact for this application is as follows:

Name: Ms Elizabeth Bruna
Email: elizabeth.bruna@gstt.nhs.uk

Who should I contact for further information?
Please do not hesitate to contact me for assistance with this application. My contact details are below.

Your IRAS project ID is **217448**. Please quote this on all correspondence.

Yours sincerely

Thomas Fairman
HRA Assessor

Email: hra.approval@nhs.net

Copy to: *Ms Elizabeth Bruna, Guy's & St Thomas' NHS Foundation Trust, (Sponsor Contact)*
Ms Jennifer Boston, Guy's & St Thomas' NHS Foundation Trust, (Lead NHS R&D Contact)

IRAS project ID	217448

List of Documents

The final document set assessed and approved by HRA and HCRW Approval is listed below.

Document	*Version*	*Date*
Copies of advertisement materials for research participants [Webpage Advertisement Text - V1.0 25-07-2017]	1.0	25 July 2017
Covering letter on headed paper [Cover Letter]	1.0	09 August 2017
Covering letter on headed paper		10 May 2018
GP/consultant information sheets or letters [GP Consultant Information Sheet or Letter]	1.0	09 August 2017
HRA Schedule of Events	1.0	06 June 2018
HRA Statement of Activities	1.0	06 June 2018
Instructions for use of medical device [User manual device]	Doc ZS-P-08-D	
Interview schedules or topic guides for participants [Interview Schedules]	1.0	09 August 2017
IRAS Application Form [IRAS_Form_06032018]		06 March 2018
Letter from funder [Letter from Funder]		10 November 2017
Letter from sponsor [R&D Approval Prof Charles Wolfe (Director of R&D GSTT) and Prof Lance McCracken (R&D Lead PCCP)]		30 March 2017
Letter from statistician [Statistician's Input]	1.0	09 August 2017
Letters of invitation to participant	1.1	10 May 2018
MHRA Notice of No Objection Letter (Medical Devices) and relevant correspondence [MHRA >>letter of no objection/email<<]	MHRA ref: E/2017/0126	10 February 2017
Other [Visual Analogue Score]		
Participant consent form	1.1	10 May 2018
Participant information sheet (PIS)	1.1	10 May 2018
Research protocol or project proposal	1.1	14 May 2018
Summary CV for Chief Investigator (CI) [CV Chief Investigator]	1.0	16 May 2017
Summary, synopsis or diagram (flowchart) of protocol in non technical language [Summary Non-Expert]	1.0	09 August 2017
Validated questionnaire [Epworth Sleepiness Scale]	1.0	04 July 2017
Validated questionnaire [EQ-5D-5L]		
Validated questionnaire [Stanford Sleepiness Scale]	1.0	04 July 2017
Validated questionnaire [Hospital Anxiety and Depression Scale]	1.0	04 July 2017
Validated questionnaire [Functional Outcomes Sleep Questionnaire]	1.0	04 July 2017

Summary of assessment

The following information provides assurance to you, the sponsor and the NHS in England and Wales that the study, as assessed for HRA and HCRW Approval, is compliant with relevant standards. It also provides information and clarification, where appropriate, to participating NHS organisations in England and Wales to assist in assessing, arranging and confirming capacity and capability.

Assessment criteria

Section	Assessment Criteria	Compliant with Standards	Comments
1.1	IRAS application completed correctly	Yes	No comments
2.1	Participant information/consent documents and consent process	Yes	No comments
3.1	Protocol assessment	Yes	No comments
4.1	Allocation of responsibilities and rights are agreed and documented	Yes	There are two site types participating in the study **Research Sites** and **PIC sites.**<br>**Research Sites**<br>Only 1 NHS organisation will participate in this study as a research site and this organisation is also the study sponsor. No agreements are therefore expected.<br>**Site Type 2**<br>The sponsor has submitted a Statement of Activities for this site type but does not intend to use this as the contract between themselves and study sites.<br>The sponsor is not requesting, and does not require any additional agreements.
4.2	Insurance/indemnity arrangements assessed	Yes	Where applicable, independent contractors (e.g. General Practitioners) should ensure that the

Section	Assessment Criteria	Compliant with Standards	Comments
			professional indemnity provided by their medical defence organisation covers the activities expected of them for this research study
4.3	Financial arrangements assessed	Yes	External study funding has been secured from the British Heart Foundation.
5.1	Compliance with the Data Protection Act and data security issues assessed	Yes	No comments
5.2	CTIMPS – Arrangements for compliance with the Clinical Trials Regulations assessed	Not Applicable	No comments
5.3	Compliance with any applicable laws or regulations	Yes	No comments
6.1	NHS Research Ethics Committee favourable opinion received for applicable studies	Yes	No comments
6.2	CTIMPS – Clinical Trials Authorisation (CTA) letter received	Not Applicable	No comments
6.3	Devices – MHRA notice of no objection received	Not Applicable	No comments
6.4	Other regulatory approvals and authorisations received	Not Applicable	No comments

Participating NHS Organisations in England

This provides detail on the types of participating NHS organisations in the study and a statement as to whether the activities at all organisations are the same or different.

The Chief Investigator or sponsor should share relevant study documents with participating NHS organisations in England and Wales in order to put arrangements in place to deliver the study. The documents should be sent to both the local study team, where applicable, and the office providing the research management function at the participating organisation. Where applicable, the local LCRN contact should also be copied into this correspondence.

If chief investigators, sponsors or principal investigators are asked to complete site level forms for participating NHS organisations in England and Wales which are not provided in IRAS or on the HRA or HCRW websites, the chief investigator, sponsor or principal investigator should notify the HRA immediately at hra.approval@nhs.net, or HCRW at Research-permissions@wales.nhs.uk. We will work with these organisations to achieve a consistent approach to information provision.

Principal Investigator Suitability

This confirms whether the sponsor position on whether a PI, LC or neither should be in place is correct for each type of participating NHS organisation in England and the minimum expectations for education, training and experience that PIs should meet (where applicable).
**Research Sites –** A Principal Investigator should be appointed at study sites of this type.<br><br>**PIC sites –** No Principal Investigator or Local Collaborator is required at study sites of this type and the CI can take local responsibility. The researchers have indicated, in the Statement of Activities, that they wish to appoint a Local Principal Investigator.<br><br>GCP training is not a generic training expectation, in line with the HRA/HCRW/MHRA statement on training expectations.

HR Good Practice Resource Pack Expectations

This confirms the HR Good Practice Resource Pack expectations for the study and the pre-engagement checks that should and should not be undertaken
As a non-commercial study undertaken by local staff, it is unlikely that letters of access or honorary research contracts will be applicable, except where local network staff employed by another Trust (or University) are involved (and then it is likely that arrangements are already in place).<br><br>Where arrangements are not already in place, network staff (or similar) undertaking any of the research activities listed in A18 or A19 of the IRAS form (except for administration of questionnaires or surveys), would be expected to obtain an honorary research contract from one NHS organisation (if university employed), followed by Letters of Access for subsequent organisations. This would be on the basis of a Research Passport (if university employed) or an NHS to NHS confirmation of pre-engagement checks letter (if NHS employed). These should confirm enhanced DBS checks, including appropriate barred list checks, and occupational health clearance.<br><br>For research team members only administering questionnaires or surveys, a Letter of Access based on standard DBS checks and occupational health clearance would be appropriate.

Other Information to Aid Study Set-up

This details any other information that may be helpful to sponsors and participating NHS organisations in England to aid study set-up.
The applicant has indicated that they do intend to apply for inclusion on the NIHR CRN Portfolio.
