# Supplementary material for: Domiciliary transcutaneous electrical stimulation in patients with obstructive sleep apnoea and limited adherence to continuous positive airway pressure therapy: a single-centre, open-label, randomised, controlled phase III trial
Source: eClinicalMedicine. 2023 Aug 3;62:102112. doi: 10.1016/j.eclinm.2023.102112 (PMC10466238; doi:10.1016/j.eclinm.2023.102112)
Supplement: Supplementary Figs. S1–S3 and Tables S1 and S2 [file mmc2.doc]

| **Full title of trial** | **Randomised controlled trial of domiciliary transcutaneous electrical stimulation in obstructive sleep apnoea: TESLA-home** |
| --- | --- |
| **Short title** | TESLA home |
| **Version and date of Clinical Investigation Plan (CIP)** | Version 1.3, 22/11/2020; IRAS 217448 |
| **Revision History** | N/A |
| **Sponsor:** |  |
| **Sponsor CIP number** | Guy’s & St Thomas’ NHS Foundation Trust V1.8 |
| **Funder (s) :** | British Lung Foundation  Channel Scheme / Egyptian Educational Office |
| **Phase of trial** | Phase III |
| **Sites(s)** | Single site |
|  |  |
| **Chief investigator:**  Name: Joerg Steier  Address: Lane Fox Unit / Sleep Disorders Centre, Guy’s and St Thomas’ NHS Foundation Trust, Westminster Bridge Road, London, SE1 7EH  Telephone: 0207 188 3434  Fax: 0207 188 6116  Email: [joerg.steier@gstt.nhs.uk](mailto:joerg.steier@gstt.nhs.uk) | **Sponsor Representative**:  Jennifer Boston  Guy's & St Thomas' Foundation NHS Trust  R&D Department  16^th^ Floor, Tower Wing  Great Maze pond  London SE1 9RT  Ext Tel: 02071887188 ext 54426  Int tel:  54426  Fax: 02071883472  [R&D@gstt.nhs.uk](mailto:R%26D@gstt.nhs.uk) |

# Signatures

The Chief Investigator and R&D (Sponsor office) have discussed this Clinical Investigation Plan (CIP). The investigators agree to perform the investigations and to abide by this CIP.

The investigator agrees to conduct the trial in compliance with the approved CIP, EU GCP and UK Regulations for Devices, the UK Data Protection Act (1998), the Trust Information Governance Policy (or other local equivalent), the Research Governance Framework (2005’ 2^nd^ Edition; as amended), the Sponsor’s SOPs, and other regulatory requirements as amended.

| **Chief investigator**  Joerg Steier |  |  |
| --- | --- | --- |
|  | Signature | Date |
| **Sponsor Representative**  R&D to Add |  |  |
| GSTFT | Signature | Date |
|  |  |  |

**This CIP template is intended for use with UK sites only.**

Contents

[Title Page 1](#__RefHeading___Toc476061611)

[Signatures 2](#__RefHeading___Toc476061611)

[Table of Content 3](#__RefHeading___Toc476061611)

[Abbreviations 5](#__RefHeading___Toc476061611)

[1 Trial Personnel 6](#__RefHeading___Toc476061612)

[2 Synopsis of Clinical Investigation 7](#__RefHeading___Toc476061613)

[3 Identification and Description of the Investigational Device 9](#__RefHeading___Toc476061614)

[4 Justification for the design of the clinical investigation 15](#__RefHeading___Toc476061616)

[5 Risks and benefits of the Investigational device and clinical Investigation 16](#__RefHeading___Toc476061617)

[6 Objectives and hypotheses of the clinical investigation 17](#__RefHeading___Toc476061619)

[6.1 Objectives, primary and secondary. 17](#__RefHeading___Toc476061620)

[6.2 Hypotheses, primary and secondary, to be accepted or rejected by statistical data from the clinical investigation. 18](#__RefHeading___Toc476061621)

[6.3 Claims and intended performance of the investigational device that are to be verified…. 18](#__RefHeading___Toc476061622)

[6.4 Risks and anticipated adverse device effects that are to be assessed. 19](#__RefHeading___Toc476061623)

[7 Design of the clinical investigation 20](#__RefHeading___Toc476061624)

[7.1 General (to include the following) 20](#__RefHeading___Toc476061625)

[7.2 Investigational device and comparators (to include the following) 20](#__RefHeading___Toc476061626)

[7.3 Subject Selection 21](#__RefHeading___Toc476061627)

[7.3.1 Inclusion criteria 21](#__RefHeading___Toc476061628)

[7.3.2 Exclusion criteria 21](#__RefHeading___Toc476061629)

[7.3.3 Criteria and procedures for subject withdrawal or discontinuation. 22](#__RefHeading___Toc476061630)

[7.3.4 Subject Enrolment (to include the following) 22](#__RefHeading___Toc476061631)

[7.4 Procedures 22](#__RefHeading___Toc476061632)

[7.4.1 Informed Consent 22](#__RefHeading___Toc476061633)

[7.4.2 Screening and Eligibility Assessment 23](#__RefHeading___Toc476061634)

[7.4.3 Baseline Assessments 24](#__RefHeading___Toc476061635)

[7.4.4 Randomisation 24](#__RefHeading___Toc476061636)

[7.4.5 Subsequent assessments 24](#__RefHeading___Toc476061637)

[7.5 Definition of End of Trial 25](#__RefHeading___Toc476061638)

[7.6 Discontinuation/ Withdrawal of Participants from Study Treatment 26](#__RefHeading___Toc476061639)

[7.7 Source Data 26](#__RefHeading___Toc476061640)

[8 Statistical Considerations 26](#__RefHeading___Toc476061641)

[9 Data Management 27](#__RefHeading___Toc476061642)

[9.1 Data to be collected 27](#__RefHeading___Toc476061643)

[9.2 Data handling and record keeping 27](#__RefHeading___Toc476061644)

[10 Deviations from clinical investigation plan 27](#__RefHeading___Toc476061645)

[11 Device accountability 28](#__RefHeading___Toc476061646)

[12 Statements of compliance 30](#__RefHeading___Toc476061647)

[12.1 Declaration of Helsinki, International Standards and national regulations 30](#__RefHeading___Toc476061648)

[12.2 Approvals 30](#__RefHeading___Toc476061649)

[12.3 Funding and Insurance 30](#__RefHeading___Toc476061651)

[13 Monitoring 30](#__RefHeading___Toc476061652)

[13.1 Confidentiality 31](#__RefHeading___Toc476061653)

[13.2 Record keeping and archiving 31](#__RefHeading___Toc476061654)

[14 Adverse events, adverse device effects and device deficiencies 32](#__RefHeading___Toc476061655)

[14.1 Definitions 33](#__RefHeading___Toc476061656)

[14.2 Safety Reporting requirements and timelines 33](#__RefHeading___Toc476061661)

[14.3 Assessments of adverse events 35](#__RefHeading___Toc476061662)

[14.3.1 Severity 35](#__RefHeading___Toc476061663)

[14.3.2 Seriousness 35](#__RefHeading___Toc476061664)

[14.3.3 Causality 35](#__RefHeading___Toc476061665)

[14.3.4 Expectedness 35](#__RefHeading___Toc476061666)

[14.4 Procedures for recording and reporting Adverse Events and Device Deficiencies 36](#__RefHeading___Toc476061667)

[14.4.1 Investigator responsibilities 36](#__RefHeading___Toc476061668)

[14.5 Reporting of all Adverse Events and Device Deficiencies: Investigator and Sponsor responsibilities 36](#__RefHeading___Toc476061669)

[14.5.1 Annual Safety Reports 37](#__RefHeading___Toc476061670)

[14.5.2 Progress reports 37](#__RefHeading___Toc476061671)

[14.6 Foreseeable adverse events and anticipated adverse device effects 37](#__RefHeading___Toc476061672)

[15 Vulnerable population 37](#__RefHeading___Toc476061675)

[16 Suspension or premature termination of the clinical investigation 37](#__RefHeading___Toc476061676)

[17 Publication policy 37](#__RefHeading___Toc476061679)

[18 Bibliography 38](#__RefHeading___Toc476061680)

[19 Appendix A: Schedule of Investigations 39](#__RefHeading___Toc476061680)

List of abbreviations

Commonly used abbreviations

AE Adverse event

ADE Adverse Device Effect

AHI Apnoea-Hypopnoea-Index

CI Chief Investigator

CPAP Continuous Positive Airway Pressure

CRF Case Report Form

CRO Contract Research Organisation

CT Clinical Trials

CTA Clinical Trials Authorisation

ESS Epworth Sleepiness Scale

GCP Good Clinical Practice

GP General Practitioner

HRA Health Research Authority

ICF Informed Consent Form

ICH International Conference of Harmonisation

IEC Independent Ethics Committee

IRB Independent Review Board

MHRA Medicines and Healthcare products Regulatory Agency

NHS National Health Service

NRES National Research Ethics Service

OSA Obstructive Sleep Apnoea

ODI Oxygen Desaturation Index

PI Principal Investigator

PIL Participant/ Patient Information Leaflet

R&D NHS Trust R&D Department

REC Research Ethics Committee

SAE Serious Adverse Event

SADE Serious Adverse Device Effect

SIL Subject Information Leaflet (see PIL)

SOP Standard Operating Procedure

TMF Trial Master File

UADE Unanticipated Adverse Device Effect

# Trial Personnel

| Chief Investigator (CI)  [Consultant] | Joerg Steier e-mail: [Joerg.steier@gstt.nhs.uk](mailto:Joerg.steier@gstt.nhs.uk)  tel: 02071883434 |
| --- | --- |
| Principal Investigator (PI)  [Consultant] | Nicholas Hart e-mail:[Nicholas.hart@gstt.nhs.uk](mailto:Nicholas.hart@gstt.nhs.uk)  tel: 02071883434 |
| Co-Investigator  [Consultant] | Michael I Polkey (Brompton) e-mail: [m.polkey@rbht.nhs.uk](mailto:m.polkey@rbht.nhs.uk)  tel: 0207 352 8121 |
| Co-Investigator  [Consultant]  Co-Investigator  [Consultant] | Kai Lee (King’s) e-mail: [kai.lee@nbhs.uk](mailto:kai.lee@nbhs.uk)  tel: 02032999000 x2080  John Moxham (KCL)  e-mail: [john.moxham@kcl.ac.uk](mailto:kai.lee@nbhs.uk)  tel: 02032999000 x3165 |
| Sponsor’s representative | Jennifer Boston e-mail: [Jennifer.boston@gstt.nhs.uk](mailto:Jennifer.boston@gstt.nhs.uk)  tel: 02071887188 ext 54426  fax: 02071883472 |
| Statistician | Abdel Douiri (KCL)  e-mail: [abdel.douiri@kcl.ac.uk](mailto:abdel.douiri@kcl.ac.uk)  tel: 02078488224 |

# Synopsis of Clinical Investigation

| **Title:** | **Randomised controlled trial of domiciliary transcutaneous electrical stimulation in obstructive sleep apnoea: TESLA-home V1.1 14/05/2018** |
| --- | --- |
| **Short title:** | TESLA home |
| **IRAS ID:** | 217448 |
| **REC Reference:** | 18/LO/0638 |
| **Device:** | PREMIER PLUS easy rechargeable TENS (TENS+, Stockport, UK) |
| **Phase of trial:** | III |
| **Objectives:** | Primary: To assess the efficacy of transcutaneous electrical stimulation of the pharyngeal dilator muscles in patients with obstructive sleep apnoea in the community compared to usual care, and follow up for 3 months.  Secondary: To assess compliance of non-invasive electrical stimulation of the upper airway dilator muscles in obstructive sleep apnoea patients over time and evaluate the control of symptoms and improvement in quality of life. |
| **Type of trial:** | Prospective, interventional, randomized, controlled, two parallel arm trial |
| **Trial design and methods:** | Following randomisation into intervention group and usual care at baseline patients will be assessed followed up at 3-months. Outcome parameters will compare improvement in the apnoea-hyopnoea index (AHI), sleepiness and quality of life at 3-months against baseline between the two groups. |
| **Trial duration per participant:** | Three months |
| **Estimated total trial duration:** | Three years |
| **Planned trial sites:** | Single site (Guy’s & St Thomas’ NHS Foundation Trust) with Royal Brompton & Harefield NHS Foundation Trust and King’s College Hospital NHS Foundation Trust as PIC sites. |
| **Total number of participants planned:** | n=68 |
| **Main inclusion/exclusion criteria:** | Patients with obstructive sleep apnoea who have low CPAP adherence will be included. Exclusion criteria are very severe OSA, morbid obesity and enlarged neck circumference. |
| **Statistical methodology and analysis:** | Statistical analysis will compare the respective outcome parameters (primary outcome: AHI; secondary outcome: ESS, ODI, compliance in hours usage/night, comfort) between active stimulation and the control group. Full statistical description will be provided and for each of the variables analysed, univariate descriptive statistics will present an overview of the data. Continuous variables will be presented as median and interquartile range (IQR), unless otherwise stated. For categorical variables, frequency counts and percentages will be presented as summary statistics for the subgroups of interest. To compare study groups, we will use the Wilcoxon and paired t-test for continuous paired variables, and the χ^2^ test for categorical variables. The primary outcome parameters (AHI) will be analysed using the Mann-Whitney test between treatment groups (active stimulation) and control group (CPAP). Similar analyses will be conducted for secondary outcomes. |

# Identification and Description of the Investigational Device

*Background*

Obstructive sleep apnoea is highly prevalent (1), with the obesity epidemic relentlessly increasing the problem (2, 3). The best treatment for uncomplicated and moderate-severe OSA remains CPAP therapy (4, 5). However, long-term adherence to treatment with CPAP is poor (6) and there are few alternatives. Electrical stimulation of the dilator muscles of the upper airway has been shown to reduce nocturnal apnoeas significantly using an invasive approach, hypoglossal nerve stimulation (7), as well as by non-invasive transcutaneous stimulation (8, 9). Although non-invasive transcutaneous stimulation may not be as effective as CPAP therapy in many OSA patients it could be an important option to treat some of the large number of patients who fail CPAP (10) and might also be of use in those whose OSA is not judged sufficiently severe to warrant CPAP.

We propose a study to use a transcutaneous electrical stimulation device in selected patients with OSA who do not tolerate CPAP therapy to treat them over a period of 3-months in the community and compare the results against usual care, ongoing CPAP therapy. The results of this study will help us to prepare a definitive multi-centre randomized controlled trial.

The primary aim of this study is to assess efficacy and generate an accurate sample size estimation, test the subjective willingness of patients to use and objectively determined compliance with the treatment over a long period, as well as to specify the drop-out rate. The improvement in sleep apnoea severity will be primary outcome measures. The feasibility of the method, including compliance, comfort, adverse events, and sleepiness will be the secondary outcome measure.

*Electrical Stimulation*

The group of patients receiving continuous transcutaneous electrical stimulation will be trained on the device and settings will be recorded. The device will be hooked on a hydrogel patch which is placed in the submental area leaving the bony structure of the mandible free. Ideally, men should shave daily to guarantee a good contact of the hydrogel and low skin resistance to promote good transduction of the current and avoid skin discomfort. In order to allow for maximal adhesion and contact the hydrogel should be placed on dry skin. The device needs to be started by pressing the “on” button. Once the device is turned on the current intensity can be increased by pushing the ‘+’ button and lowered by pushing the ‘-‘ button. When a comfortable skin sensation is felt the patients should press the ‘-‘ button once more. At that time the device stimulates with a low current which is not felt but provides a neuromuscular tone to the muscles whilst asleep. The device is kept on all night and in the morning taken off with the hydrogel and disconnected. Once disconnected it should be turned off by pressing the “off” button. The device should then be placed on the provided charger so that it is fully charged again for the next night.

Advice on sleep hygiene, posture in bed and general life style (the use of alcohol, sedatives) will be discussed, the potential impact of weight on OSA will be explained. Weekly phone calls and follow up visits at 6- and 12-weeks will be organized. At each visit comfort, compliance and adverse reactions will be recorded. At 12-weeks, the patients will be invited for a repeat assessment including polysomnography during a night of electrical stimulation. Usage time of the device will be discussed with the patients and recorded.

*General Description*

The device works by passing mild electrical signals into the body via a disposable adhesive gel pad on the skin and stimulates muscles to contract, in particular those associated with opening the airways (genioglossus muscles). Application of regular electrical pulses to these muscles, which start after the user has fallen asleep, help to maintain more open airways. These muscles might otherwise be too relaxed and cause vibrations in the throat whilst breathing, manifesting as snoring or sleep apnoea.

| 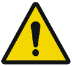 **Warning:** | Before you start it is vital to read, understand, and strictly observe all warnings, cautions, contraindications, notes, and safety markings within this document and on the product |
| --- | --- |

| 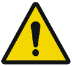  **Warning:** | **Warning:**   - Ensure that the gel pads are correctly attached to the product and the correct area of the body. - Only use electrode pads provided. - Do not use with skin products, topically applied creams or ointments. - Avoid contact of the gel pads with any metallic objects, such as necklaces. - Electrode pads are for limited use, repeated use on the skin may increase the risk of irritation and infections. - Do not use on a wet body. - Keep device dry, do not bath or shower whilst wearing the device. - Do not use in the bathroom or in any other area of high humidity as this may cause uncomfortable levels of stimulation. - Apply device to intended area only. |
| --- | --- |

| 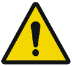  **Warning:** | Do not attempt to wear the device whilst charging. |
| --- | --- |
| 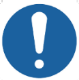 **Caution:** | This device is not waterproof |
| 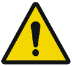  **Warning:** | Electrode pads are for limited use, repeated use on the skin may increase the risk of irritation and infections. |

Periodically clean device with a damp cloth or alcohol-based wipe. Do not immerse in cleaning fluid, allow to air-dry before charging or use. If required, clean disposable electrode pad with a damp cloth only. Avoid extensive cycles of cleaning and reuse on electrode pad, repeated use on the skin may increase the risk of irritation and infections. Replace protective film to prevent pad from drying out.

*Contraindications of Use*

| 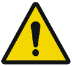 | **Warning:** Do not use this device if any contraindication is applicable, if in doubt consult a medical professional. |
| --- | --- |

- Do not use on individuals with pacemakers, defibrillators, or any other implantable medical devices, including but not limited to hyperglossal nerve stimulators, or cerebral shunts.
- Do not use near sources of enriched oxygen.
- Do not use with metal implants (excluding fillings) in the head and neck region.
- Do not use following recently diagnosed deep vein thrombosis (DVT).
- Do not use if diagnosed with cancer, do not apply over or in proximity to cancerous lesions.
- Do not use if suffering from migraine.
- Do not use if pregnant or in labour.
- Do not use if suffering from heart conditions, arrhythmias, recurrent seizures, epilepsy, alcoholism, leprosy.
- Do not use following surgery where muscle contractions may disrupt the healing process.
- Do not use on scarred areas following surgery for at least 10 months after operation.
- Do not use when connected to high frequency surgical equipment.
- Do not use in conjunction with any other medical devices, including but not limited to: TENS machine, electrical stimulation machine, electrocardiograph (ECG) meter, Magnetic Resonance Imaging (MRI), and electronic diagnosis scanners (e.g. CT scanner).
- Seek medical advice prior to use for users suffering from severe mental illness, Alzheimer’s, dementia, or any other memory impairment.
- Seek medical advice prior to use if suffering from: abnormal blood pressure, skin conditions including broken or damaged skin, loss of feeling in areas of the body, diabetes and acute disease/fevers.
- Do not apply to swollen, infected, inflamed areas or broken skin.
- In some cases, skin inflammation or irritation can develop in the contact area. Stop using the device immediately if you notice any reddening of the skin, if you feel any discomfort or feel unwell (seek medical advice).

*General Warnings and Cautions*

| 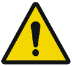 | **Warning:** Not to be used for children under 16. |
| --- | --- |
| 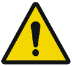 | **Warning:** Keep out of reach of children and pets. |
| 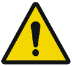 | **Warning:** Do not apply to the head, face, eyes, mouth, oral cavity, limbs, sexual organs, torso, spine, over bones, or sides of the throat and neck (i.e. over thyroid or carotid sinus regions). |
| 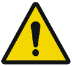 | **Warning:** Users with an implanted electronic device, such as a pacemaker, should not use this product without consulting their medical professional. |
| 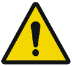 | **Warning:** Simultaneous connection of a patient to a h.f. surgical equipment may result in burns at the site of the stimulator electrodes and possible damage to the stimulator. |
| 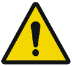 | **Warning:** Operation in close proximity (e.g. 1m) to a short wave or microwave therapy equipment may produce instability in the stimulator output. |
| 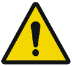 | **Warning:** Application of electrodes near the heart could increase the risk of cardiac fibrillation. |
| 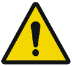 | **Warning:** Do not use whilst driving, operating machinery, or during any activity where involuntary muscle contractions may put the user or others at risk. |
| 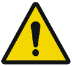 | **Warning:** Do not use if packaging is open or appears damaged. |
| 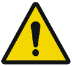 | **Warning:** Check the device is functioning correctly. |
| 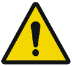 | **Warning:** Do not switch on until correctly attached. |
| 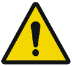 | **Warning:** The device is intended to be used for a maximum of 9 hours. |

# Justification for the design of the clinical investigation

Obstructive sleep apnoea (OSA) is the most common form of sleep-disordered breathing (1-3). The prevalence has significantly increased over the last two decades due to the obesity epidemic and OSA affects now roughly 10% of the middle aged male population (2), leaving some populations with difficulties to find enough sleep services within their area (3). Untreated OSA leads to adverse long term health outcomes, including cardiovascular risks (11) including stroke, and excessive daytime sleepiness. The best available treatment for moderate-severe OSA remains CPAP therapy (4), which is currently recommended by NICE (5). The need to sleep with a mask impacts on long-term compliance with treatment and contributes to the low long-term uptake of CPAP (6) leaving few alternatives (12). Mandibular advancement devices, although used in the treatment of mild OSA, are not sufficient for the long-term therapy of moderate-severe OSA, which is reflected in current guidance (13).

The need to develop additional alternatives to CPAP therapy has recently led to a renaissance of electrical stimulation (10). The stimulation of upper airway dilator muscles, invasively via hypoglossal nerve stimulation (7, 14, 15) and non-invasively via transcutaneous electrical stimulation (8, 9) has attracted considerable interest (16). Hypoglossal nerve stimulation does not seem to be as efficient as CPAP therapy, but in a recent analysis of published data positive studies report an improvement in the apnoea-hypopnoea-index (AHI) or the oxygen-desaturation-index (ODI), both markers of the severity of OSA, of between 50-75% (7, 10). In untreated patients with OSA (in whom CPAP was not tolerated), this effect size would be sufficient to downstage severe OSA, as defined by an AHI or ODI above 30 events per hour, to a milder category (mild OSA <15 events per hour), which could still cause symptoms like sleepiness but would be likely to be associated with no or minimal cardiovascular long-term risks (11).

Following our initial pilot study using non-invasive electrical stimulation for short periods at night (8) we have studied the feasibility of this method for whole night use in a randomized sham-controlled cross-over trial with a prototype device at the bedside in the sleep laboratory (NCT01661712), the TESLA trial (9). This trial revealed that there was a modest improvement in sleep apnoea in the studied cohort with some patients being identified as responders and some as non-responders. 17/36 patients had a clinically relevant improvement, as defined by a more than 25% improvement in the oxygen desaturation index (4%ODI) and/or a 4%ODI of less than 5/hour on treatment (9).

In order to establish whether transcutaneous electrical stimulation can be used to treat sleep apnoea in the responding cohort in the domiciliary setting a medical device has been developed for use in a domiciliary study. We propose to recruit suitable patients with obstructive sleep apnoea and provide the treatment at home over a three-month period; patients would need to use transcutaneous electrical stimulation every night when asleep. The focus of this study is on efficacy, as assessed by the sleep apnoea severity and symptomatic improvement, patient compliance, as measured by usage (total hours / per night), and on subjective comfort and acceptance. The results of this study would be used to inform us about sample size generation and recruitment for a later multi-centre trial (Phase III) of this method.

# Risks and benefits of the Investigational device and clinical Investigation

*General Benefit*

The proposed study will provide us with information about the efficacy of domiciliary treatment with electrical stimulation, the number of eligible patients and data to perform a sample size analysis for a definitive and prospective multi-centre randomized controlled trial (Phase III). The study design will test the willingness of patients for randomisation, it will reveal whether clinicians refer patients to participate in this study, and the follow up of patients on continuous transcutaneous electrical stimulation (CTES) over a period of three months will test retention in the study. We will get an understanding whether the proposed outcome parameters, the AHI and 4%ODI, the ESS, as well as compliance and comfort are suitable measures and whether the collection of data, questionnaires and time to collect the data are within reasonable acceptance for the patients and the study team, as well as genioglossus thickening, as determined by ultrasound.

*Specific Risks and Benefits*

The patient will be given a printed information sheet informing them of potential benefits and risks of the trial on the day of their outpatient clinics appointment. They will be explained the treatment options for sleep apnoea. It will then be explained what intervention is planned in the trial. The potential participant will be contacted >24h later to be given time to reconsider, ask friends, family members and

GP`s about this trial and come to a decision. If the potential participant agrees to be included in the trial they will be invited to come back and informed written consent will be taken following sufficient time to ask further questions. Participants will be screened and treated for obstructive sleep apnoea. The transcutaneous electrical stimulation device will be introduced to patients as a new treatment that they may be able to choose at a later stage. Electrical stimulation with transcutaneous patches, similar to those used in transcutaneous electrical nerve stimulation (TENS) machines, is widely and safely used without significant side effects reported. Hypothetically, high electrical current can lead to discomfort on the skin and muscle spasm but the titration of the current according to patient perception will help to minimise these effects.

We recently published results from a randomised controlled trial using a similar medical device to the one we propose to use in this trial and found that patients tolerated the electrical stimulation well (Pengo M et al, THORAX 2016). Patients did not report any local skin reaction, nor any unpleasant or painful sensation on the skin. There were no adverse effects when using the device.

However, we acknowledge that the new device that we are proposing to use in this trial differs from the TENS machine that we used in the above trial. The current device will be used every night for three months, as opposed to a one-off treatment. Therefore, we have the following procedures in place to avoid problems:

The group of patients receiving continuous transcutaneous electrical stimulation will be trained on the device and settings will be recorded. The device will be hooked on a hydrogel patch which is placed in the neck area leaving the bony structure of the mandible free. The device can only be charged when off the body. Ideally, men should shave daily to guarantee a good contact of the hydrogel and a low skin resistance to promote good transduction of the current and avoid skin discomfort. In order to allow for maximal adhesion and contact the hydrogel should be placed on dry skin. The device needs to be started by pressing the ‘on’ button. Once the device is turned on the current intensity can be increased by pushing the ‘+’ button and lowered by pushing the ‘-‘ button. When a comfortable skin sensation is felt the patients should press the ‘-‘ button once more. At that time the device stimulates with a low current which is not felt but will maintain a neuromuscular tone to the muscles whilst asleep. The device is kept on all night and in the morning is taken off together with the hydrogel and disconnected. Once disconnected it should be turned off by pressing the ‘off’ button. The device should then be placed on the provided charger so that it is fully charged again for the next night.

Advice on sleep hygiene, posture in bed and general life style (the use of alcohol, sedatives) will be discussed, the potential impact of weight on OSA will be explained. Weekly phone calls and follow up visits at 6- and 12-weeks will be organized. At each visit comfort, compliance and adverse reactions will be recorded. At 12-weeks, the patients will be invited for a repeat assessment including polysomnography during a night of electrical stimulation in the sleep laboratory. Usage time of the device will be discussed with the patients and recorded.

We will discuss the following instructions (see below) in detail with the patient, in order to ensure they understand how to use the device, and what action to take in case of a safety issue.

# Objectives and hypotheses of the clinical investigation

*General Objectives and Hypotheses*

The objective is to assess efficacy and compliance of transcutaneous electrical stimulation of the upper airway dilator muscles in obstructive sleep apnoea patients over time and evaluate acceptability and comfort. The proposed study will provide us with information about the efficacy of domiciliary treatment, the number of eligible patients and data to perform a sample size analysis for a definitive and prospective multi-centre randomized controlled trial (Phase III). The study design will test the willingness of patients for randomisation, it will reveal whether clinicians refer patients to participate in this study, and the follow up of patients on continuous transcutaneous electrical stimulation (CTES) over a period of three months will test retention in the study. We will get an understanding whether the proposed outcome parameters, the AHI and 4%ODI, the ESS, as well as compliance and comfort are suitable measures and whether the collection of data, questionnaires and time to collect the data are within reasonable acceptance for the patients and the study team, as well as genioglossus thickening, as determined by ultrasound.

## Objectives, primary and secondary.

The *primary outcome* parameter will be the reduction in severity of OSA at 12-weeks compared to baseline, as defined by the AHI. A response to treatment will be a more than 50% reduction in the AHI from baseline and a total AHI< 20/h, in line with previous trials (7, 9).

*Secondary outcome* parameters will address the ESS, the 4%ODI, compliance with treatment, as measured by the usage of transcutaneous electrical stimulation at night (total hours / per night). Subjective comfort and acceptance will be assessed using a visual analogue scale (0 – 10 points), the disease-specific symptoms, as assessed by sleep-related Quality of Life (FOSQ, EQ-5D), as well as snoring (total duration, intensity, spouse reporting). Ultrasound will be used to assess genioglossus thickness and preserved contractibility.

## Hypotheses, primary and secondary, to be accepted or rejected by statistical data from the clinical investigation.

Statistical analysis will compare the respective outcome parameters (primary outcome: AHI; secondary outcome: ESS, 4%ODI, compliance in hours usage/night, comfort) between active stimulation and the control group. Full statistical description will be provided and for each of the variables analysed, univariate descriptive statistics will present an overview of the data. Continuous variables will be presented as median and interquartile range (IQR), unless otherwise stated. For categorical variables, frequency counts and percentages will be presented as summary statistics for the subgroups of interest. To compare study groups, we will use the Wilcoxon and paired t-test for continuous paired variables, and the χ^2^ test for categorical variables. The primary outcome parameters (AHI) will be analysed using the Mann-Whitney test between treatment groups (active stimulation) and control group (CPAP). Similar analyses will be conducted for secondary outcomes. The trial follows up each patient for at least 12 weeks and some patients will be inevitably lost to follow-up. Sample size estimation assumed 20% of patients would not provide end of study information that could be evaluated. If this rate is observed, data for many patients will be only partially observed. For patients who withdraw or drop out before the end of the study, the “no change assumption” will be used to impute the missing subsequent values. This may introduce a bias if the main reason for drop-out was deterioration. To examine this possibility, sensitivity analysis will be performed to assess the primary efficacy outcome using different imputation methods including “best-case scenario”, and “worst- case scenario” possible scores for the missing data as well as Markov Chain Monte Carlo Multiple Imputation. A second per protocol analysis, including only those subjects with complete follow-up data will also be performed. All analyses will be performed using StataTM version 12. Differences will be considered significant at p<0.05.

## Claims and intended performance of the investigational device that are to be verified

The Zeus device is a portable, battery powered apparatus for treatment of snoring or sleep apnoea, comprising:

- A pair of electrodes, and
- A power supply that generates an electrical signal between the electrodes to stimulate the patients’s tongue muscle (genioglossus) when the electrodes are attached to the patients.
- The device will be used in patients with obstructive sleep apnoea and compared to usual care, continuous positive airway pressure (CPAP) therapy.

## Risks and anticipated adverse device effects that are to be assessed.

The patient will be given a printed information sheet informing them of potential benefits and risks of the trial on the day of their outpatient clinics appointment. They will be explained the treatment options for sleep apnoea. It will then be explained what intervention is planned in the trial. The potential participant will be contacted >24h later to be given time to reconsider, ask friends, family members and

GP`s about this trial and come to a decision. If the potential participant agrees to be included in the trial they will be invited to come back and informed written consent will be taken following sufficient time to ask further questions. Participants will be screened and treated for obstructive sleep apnoea. The transcutaneous electrical stimulation device will be introduced to patients as a new treatment that they may be able to choose at a later stage. Electrical stimulation with transcutaneous patches, similar to those used in transcutaneous electrical nerve stimulation (TENS) machines, is widely and safely used without significant side effects reported. Hypothetically, high electrical current can lead to discomfort on the skin and muscle spasm but the titration of the current according to patient perception will help to minimise these effects.

We recently published results from a randomised controlled trial using a similar medical device to the one we propose to use in this trial and found that patients tolerated the electrical stimulation well (Pengo M et al, THORAX 2016). Patients did not report any local skin reaction, nor any unpleasant or painful sensation on the skin. There were no adverse effects when using the device. However, we acknowledge that the new device that we are proposing to use in this trial differs from the TENS machine that we used in the above trial. The current device will be used every night for three months, as opposed to a one-off

treatment. Therefore, we have the following procedures in place to avoid problems:

The group of patients receiving continuous transcutaneous electrical stimulation will be trained on the device and settings will be recorded. The device will be hooked on a hydrogel patch which is placed in the neck area leaving the bony structure of the mandible free. The device can only be charged when off the body. Ideally, men should shave daily to guarantee a good contact of the hydrogel and a low skin resistance to promote good transduction of the current and avoid skin discomfort. In order to allow for maximal adhesion and contact the hydrogel should be placed on dry skin. The device needs to be started by pressing the ‘+’ and the ‘-‘ button together for 3s. Once the device is turned on the current intensity can be increased by pushing the ‘+’ button (8 levels) and lowered by pushing the ‘-‘ button. When a comfortable skin sensation is felt the patients should press the ‘-‘ button once more. At that time the device stimulates with a low current which is not felt but will maintain a neuromuscular tone to the muscles whilst asleep. The device is kept on all night and in the morning is taken off together with the hydrogel and disconnected. Once disconnected it should be turned off by pressing the ‘+’ and the ‘-‘ button for 3s. The device should then be placed on the provided charger so that it is fully charged again for the next night. Advice on sleep hygiene, posture in bed and general life style (the use of alcohol, sedatives) will be discussed, the potential impact of weight on OSA will be explained. Weekly phone calls and follow up visits at 6- and 12-weeks will be organized. At each visit comfort, compliance and adverse reactions will be recorded. At 12-weeks, the patients will be invited for a repeat assessment including polysomnography during a night of electrical stimulation in the sleep laboratory. Usage time of the device will be discussed with the patients and recorded.

We will discuss the following instructions (see below) in detail with the patient, in order to ensure they understand how to use the device, and what action to take in case of a safety issue.

Instructions:

Before patients start it is vital to read, understand, and strictly observe all warnings, cautions, contraindications, notes, and safety markings within this document and on the product.

Warning:

• Ensure that the gel pads are correctly attached to the product and the correct area of the body.

• Only use electrode pads provided.

• Do not use with skin products, topically applied creams or ointments.

• Avoid contact of the gel pads with any metallic objects, such as necklaces.

• Electrode pads are for limited use, repeated use on the skin may increase the risk of irritation and infections.

• Do not use on a wet body.

• Keep device dry, do not bath or shower whilst wearing the device.

• Do not use in the bathroom or in any other area of high humidity as this may cause uncomfortable levels of stimulation.

• Apply device to intended area only.

• Do not attempt to wear the device whilst charging.

The device must not be used with a cardiac pacemaker or a metal implant in situ, dental prostheses are excluded. Periodically cleaning of the device with a damp cloth or alcohol-based wipe might be required, but it should not be immersed in cleaning fluid, time should be allowed to air-dry before charging or use. If required, participants should clean the disposable electrode pad with a damp cloth only, but avoid extensive cycles of cleaning and reuse of electrode pads; repeated use on the skin may increase the risk of skin irritation and infections. Participants will be shown how to replace the protective film to prevent pad from drying out.

For any queries, there is the chance to contact a 24/7 staffed telephone line to seek advice (Lane Fox Unit 02071883434/-83435).

# Design of the clinical investigation

## General

## This is a prospective trial in a randomized controlled two-arm parallel group design testing transcutaneous electrical stimulation in obstructive sleep apnoea for use at home for a 3-months’ period and compare outcomes against usual care (CPAP). Patients will be admitted to the Lane Fox Unit / Sleep Disorders Centre at Guy’s & St Thomas’ NHS Foundation Trust for their baseline assessment. Following a diagnostic sleep study they will be randomized into transcutaneous electrical stimulation at home and usual care treatment. All patients will be followed up at 6 weeks in outpatient clinic and at 3-months’ for a repeat sleep study.

## Investigational device and comparators

*Description of Active Treatment*

The group of patients receiving continuous transcutaneous electrical stimulation will be trained on the device and settings will be recorded. The device will be hooked on a hydrogel patch which is placed in the submental area leaving the bony structure of the mandible free. Ideally, men should shave daily to guarantee a good contact of the hydrogel and low skin resistance to promote good transduction of the current and avoid skin discomfort. In order to allow for maximal adhesion and contact the hydrogel should be placed on dry skin. The device needs to be started by pressing the ‘on’ button together. Once the device is turned on the current intensity can be increased by pushing the ‘+’ button and lowered by pushing the ‘-‘ button. When a comfortable skin sensation is felt the patients should press the ‘-‘ button once more. At that time the device stimulates with a low current which is not felt but provides a neuromuscular tone to the muscles whilst asleep. The device is kept on all night and in the morning taken off with the hydrogel and disconnected. Once disconnected it should be turned off by pressing the ‘off’ button. The device should then be placed on the provided charger so that it is fully charged again for the next night.

Advice on sleep hygiene, posture in bed and general life style (the use of alcohol, sedatives) will be discussed, the potential impact of weight on OSA will be explained. Weekly phone calls and follow up visits at 6- and 12-weeks will be organized. At each visit comfort, compliance and adverse reactions will be recorded. At 12-weeks, the patients will be invited for a repeat assessment including polysomnography during a night of electrical stimulation. Usage time of the device will be discussed with the patients and recorded.

*Description of Usual Care*

Patients who will be randomized to the usual care group will be given their own CPAP device, this is the standard treatment according to guidelines. In addition, the mask will be reviewed and explained again, advice on sleep hygiene, posture in bed and general life style, as well as the potential impact of weight loss on OSA will be explained. The device will be fitted and explained in the same way as in he clinical service. Weekly phone calls and follow up visits at 6- and 12-weeks will be organized. At the last visit, the patients will be studied during a repeat inpatient polysomnography and the initial assessment will be repeated.

In order to improve recruitment and retention of patients to the usual care group all participants will be offered a free and voluntary trial of the non-invasive electrical stimulation following successful completion of the trial and prior to the regular follow up in the clinical sleep service.

## Subject Selection

Patients with obstructive sleep apnoea diagnosed by overnight polysomnography will be recruited in this trial. Obstructive sleep apnoea is defined as an AHI>5/hour plus symptoms of sleepiness (ESS>10points). The sample size calculation of n=60 patients is based on previously published data of the STAR trial (7) to detect a difference in the treatment arms with a Power of 95% and an alpha of 5% using the improvement in the AHI as an outcome parameter.

Healthy volunteers will participate according to inclusion and exclusion criteria. The sample size calculation of n= volunteers to record responses to different levels of electrical current.

### Inclusion criteria

Patients who have mild-moderate OSA (AHI 5-35/hour) will be included in this study if they fail to use or have been withdrawn from standard care (CPAP <4hours/night). We will include patients with a body mass index (BMI) of 18.5-32 kg/m^2^, without significant anatomical obstruction in the upper airway (e.g. normal sized tonsils).

Apparently healthy volunteers, with BMI of 18.5-32 kg/m^2^ and without significant anatomical obstruction in the upper airway (e.g. normal sized tonsils).

### Exclusion criteria

We will exclude patients with no (AHI <5/h) or severe obstructive sleep apnoea (AHI>30/hour), exclusively postural sleep apnoea, isolated Rapid-Eye-Movement (REM) sleep associated OSA, patients who are cachectic (BMI <18.5 kg/m^2^) or very obese (BMI >32 kg/m^2^), hypercapnic patients (pCO_2_>6.5 kPa) or those with other features of obesity hypoventilation syndrome (elevated bicarbonate, HCO3^-^ >28mmol/L). Patients should not have enlarged tonsils (size 3-4), polyps and adenoids, neuromuscular disease, hypoglossal nerve palsy, abnormal pulmonary function tests, severe pulmonary hypertension, valvular heart disease, heart failure (New York Heart Association, NYHA III-IV), myocardial infarction and significant cardiac arrhythmias, uncontrolled hypertension, active psychiatric disease, co-existing non-respiratory sleep disorder, or significant metal implants or cardiac/other pacemakers. These criteria are consistent with our experience from previous trials using electrical stimulation in OSA and likely to define responders; these criteria are also consistent with experiences from trials using hypoglossal nerve stimulation (7-9). In addition, patients will need to be excluded if they have facial hair that affects the correct placement of the hydrogel patch, if they are unwilling to shave. Patients and volunteers with allergy to plasters or similar products will be excluded.

### Criteria and procedures for subject withdrawal or discontinuation.

Sample size estimation assumed 15-20% of patients would not provide end of study information that could be evaluated. If this rate is observed, data for some patients will be only partially observed. For patients who withdraw or drop out before the end of the study, the “no change assumption” will be used to impute the missing subsequent values. This may introduce a bias if the main reason for drop-out was deterioration. To examine this possibility, sensitivity analysis will be performed to assess the primary efficacy outcome using different imputation methods including “best-case scenario” and “worst- case scenario” possible scores for the missing data. A second per protocol analysis, including only those subjects with complete follow-up data will also be performed.

### Subject Enrolment

Patients with OSA will be recruited from the Lane Fox Unit and the Sleep Disorders Centre at Guy’s & St Thomas’ NHS Foundation Trust where they are assessed in the Sleep Apnoea / CPAP clinics. The Lane Fox Unit runs daily outpatient clinics at the St Thomas’ site while the Sleep Disorders Centre has multiple clinics running Mondays-Fridays (am/pm) at Nuffield House at Guy’s. Currently, the Lane Fox Unit cares for >1,600 patients on home non-invasive ventilation and CPAP therapy while the Sleep Disorders Centre has >10,000 patients on their records using CPAP therapy.

Patients will also be identified from the sleep clinics at the Royal Brompton Hospital and King’s College Hospital (PIC sites), but the study procedures will be undertaken at Guy’s & St Thomas’ Hospital. The direct care team will approach patients after consultation with the Consultant and hand out a Patient Information Sheet to discuss the study with them. Informed written consent will be taken after sufficient time to allow for information and questioning. Patients will then be offered a date to come to hospital within four weeks for the baseling assessment with overnight polysomnography. If the patient fulfills all inclusion and no exclusion criteria, follow up will be booked at 6- and 12-weeks following randomisation. Procedure on volunteers will take place at Lane Fox Unit.

## Procedures

### Informed Consent

A member of the direct clinical care team (Good Clinical Practice, GCP-trained) will take informed consent once the patients and the volunteers have had the opportunity to read and discuss the Patient Information Sheet with sufficient time. It will be clearly stated that the participant is free to withdraw from the study at any time without this affecting any future care and with no obligation to give the reason for withdrawal.

Following sufficient time and the opportunity to question the (Co-)investigator or other independent parties to decide whether they will participate in the study written informed consent will then be obtained. A copy of the signed informed consent will be given to the participants. The original signed form will be kept at the study site and a copy will be inserted in the medical notes.

### Screening and Eligibility Assessment

Patients with OSA will be recruited from the Lane Fox Unit and the Sleep Disorders Centre at Guy’s & St Thomas’ NHS Foundation Trust where they are assessed in the Sleep Apnoea / CPAP clinics. The Lane Fox Unit runs daily outpatient clinics at the St Thomas’ site while the Sleep Disorders Centre has multiple clinics running Mondays-Fridays (am/pm) at Nuffield House at Guy’s. Currently, the Lane Fox Unit cares for >1,600 patients on home non-invasive ventilation and CPAP therapy while the Sleep Disorders Centre has >10,000 patients on their records using CPAP therapy.

Patients will also be identified from the sleep clinics at the Royal Brompton Hospital and King’s College Hospital (PIC sites), but the study procedures will be undertaken at Guy’s & St Thomas’ Hospital. The direct care team will approach patients after consultation with the Consultant and hand out a Patient Information Sheet to discuss the study with them. Informed written consent will be taken after sufficient time to allow for information and questioning. Patients will then be offered a date to come to hospital within four weeks for the baseling assessment with overnight polysomnography. If the patient fulfills all inclusion and no exclusion criteria, follow up will be booked at 6- and 12-weeks following randomisation.

### Baseline Assessments

The initial assessment will include an overnight Sleep Study (Polysomnography, PSG). Patients will be assessed using the following parameters:

a) Demographics

i. Age

ii. Gender

iii. Height, Weight, Body-Mass-Index (BMI)

iv. Neck circumference

v. Waist, Hip, W:H ratio

vi. Mallampati and Friedman Score

vii. Basic lung function (spirometry: FEV_1_, FVC)

viii. Blood pressure and heart rate

ix. Medication use

x. Number of pillows used

b) Upper Airway and Carotides

i. Mallampati and Friedman score

ii. Response to transcutaneous electrical stimulation

(visual inspection using endoscopy and ultrasound)

c) Blood tests and blood pressure measurements

i. Full blood count

ii. Renal and hepatic profile

iii. Thyroidal function tests

iv. Ferritin

v. HbA1c

vi. BNP

vii. Arterial or earlobe blood gas analysis (including pH, pO_2_, pCO_2_, HCO_3_^-^)

viii. 24-hour blood pressure measurement

d) Sleep study parameters, as measured by polysomnography

i. AHI, 4%ODI

ii. Total sleep time

iii. Sleep onset

iv. Sleep efficiency

v. Diagnosis (obstructive, central, mixed sleep apnoea)

vi. Posture in bed

vii. Different sleep stages

viii. Snoring (total duration, intensity)

ix. Neural respiratory drive (EMGpara)

e) Symptom and Quality of Life scores

i. Epworth Sleepiness Scale (ESS)

ii. Stanford Sleepiness Scale (SSS)

iii. European Quality of Life tool (EQ-5D)

iv. Hospital Anxiety and Depression Scale (HADS)

v. Functional Outcome of Sleep Questionnaire (FOSQ)

vii. Qualitative overnight report

viii. if available, spouse reporting snoring and sleep quality (Visual Analog Scales, ESS)

During the COVID-19 pandemic assessments will be remotely (via telephone or videocall, a-c) or at home (d, e). For the home sleep studies multi-channel wearable sleep technology will be used (Apnea Link or NoxT3, ResMed, UK); the equipment can be couriered out and, upon return, cleaned and quarantined to avoid any risk associated with the COVID-19 virus. The patient will be provided with a manual how to use the equipment, the correct fitting will be explained via phone / videocall, and the provider’s teaching material will be made accessible online as well (additional information for the home-based sleep study and the instruction on how to use it are provided in the attached leaflet labelled “Home Sleep Study”, dated 22-11-2020, V1.0).

### Randomisation

*Randomisation and Home Treatment*

Following the baseline assessment patients’ degree of sleep apnoea will be reviewed and, if eligible, patients will be randomized into active treatment or usual care (CPAP). The active treatment will be domiciliary transcutaneous electrical stimulation at night using a medical device while the usual care group will receive ongoing CPAP therapy.

Randomisation will involve assigning a unique patient number in sequential, ascending chronological order. This number will be a two-digit number prefixed by “R” (e.g. R01, R02 etc) and will be used to identify the treatment order the patient was randomised to. Treatment assignment will be determined according to a computer generated randomisation list prepared by an individual not directly involved in subject assessments.

### Subsequent assessments

*Bi-weekly Telephone Contacts*

All patients will receive bi-weekly phone calls to encourage usage of the device and discuss problems with the treatment. Patients will be asked about comfort and adverse events / serious adverse events. The following parameters will be assessed:

i. Epworth Sleepiness Scale (ESS)

ii. Stanford Sleepiness Scale (SSS)

iii. European Quality of Life tool (EQ-5D)

iv. Functional Outcome of Sleep Questionnaire (FOSQ)

vii. Comfort (analogue scale, 0-10 points)

*Follow up at 6-weeks*

At six weeks, patients will be followed in the outpatient clinic at the Lane Fox Unit / Sleep Disorders Centre. The following assessments will be made (for more details see above):

a) Demographics

b) Blood tests

c) Symptom and Quality of Life scores

d) Usage and compliance with medical device and treatment

i. the total usage (hours) and usage per night (hours)

ii. subjective comfort with the device (Visual Analogue Scale)

iii. comfort and AE / SAE

iv. usage of number of hydrogel patches

e) Nocturnal pulse oximetry on treatment at home (will be returned via mail)

At this stage, the patients will be educated on the device/CPAP again and encouraged to continue with its usage.

During the COVID-19 pandemic assessments will be remotely (via telephone or videocall, a-d) or at home (e). For the home sleep studies multi-channel wearable sleep technology will be used (Pulse Oximetry or Apnea Link or NoxT3, ResMed, UK); the equipment can be couriered out and, upon return, cleaned and quarantined to avoid any risk associated with the COVID-19 virus. The patient will be provided with a manual how to use the equipment, the correct fitting will be explained via phone / videocall, and the provider’s teaching material will be made accessible online as well (additional information for the home-based sleep study and the instruction on how to use it are provided in the attached leaflet labelled “Home Sleep Study”, dated 22-11-2020, V1.0).

*Follow up at 12-weeks*

At 12-week follow up the patients will be invited to attend the Lane Fox Unit / Sleep Disorders Centre for another overnight polysomnography. The assessment will repeat the following measurements (for more details see above):

i) Demographics

ii) Upper Airway

iii) Blood tests and blood pressure measurement.

iv) Sleep study parameters, as measured using a polysomnography

v) Symptom and Quality of Life scores

vi) Usage and compliance of Treatment (for more details see 6.6)

Patients will be given a debriefing by the study team and individual study reports can be requested at this stage, they will be provided once the entire analysis has been completed. Qualitative data will be recorded for a potential later multi-centre trial.

During the COVID-19 pandemic assessments will be remotely (via telephone or videocall, i-iii, v, vi) or at home (iv). For the home sleep studies multi-channel wearable sleep technology will be used (Apnea Link or NoxT3, ResMed, UK); the equipment can be couriered out and, upon return, cleaned and quarantined to avoid any risk associated with the COVID-19 virus. The patient will be provided with a manual how to use the equipment, the correct fitting will be explained via phone / videocall, and the provider’s teaching material will be made accessible online as well (additional information for the home-based sleep study and the instruction on how to use it are provided in the attached leaflet labelled “Home Sleep Study”, dated 22-11-2020, V1.0).

## Definition of End of Trial

The trial finishes with the 12-week follow up assessment. The patients will then be referred back to standard care to be followed up in the outpatient setting at the Lane Fox Unit / Sleep Disorders Centre, the Royal Brompton Hospital or King’s College Hospital, as appropriate.

## Discontinuation/ Withdrawal of Participants from Study Treatment

Each participant has the right to withdraw from the study at any time without providing a reason. In addition, the investigator may discontinue the study at any time if considered necessary for one of the following reasons:

- Ineligibility (either arising during the study or retrospectively),
- Disease progression which requires discontinuation of the study or inability to continue to comply with study procedures,
- Consent withdrawn,
- Lost to follow up.
- The reason for withdrawal will be recorded, including ‘no reason given’, in the case report forms (CRF).

## Source Data

Source documents are original documents, data, and records from which participants’ CRF data are obtained. These include, but are not limited to, hospital records (from which medical history and previous and concurrent medication and sleep apnoea treatment may be summarised into the CRF), clinical and office charts and software (e.g. Somnologica / Alice).

CRF entries will be considered source data if the CRF is the site of the original recording (e.g., there is no other written or electronic record of data). In this study the CRF will be used as the source document for stable patients recruited into the study. All documents will be stored safely on password-protected systems in locked rooms at the Lane Fox Unit Clinical Research Facility. Only the research team will have access to the data. Rooms are locked and computers are password protected. On all study-specific documents, other than the signed consent, the participant will be referred to by the study participant number/code, not by name. Data are stored for the duration of the study (3 years) and then archived (for at least another 5 years).

# Statistical Considerations

*Sample size*

We performed a sample size analysis based on the previous trial by Strollo et al  (11) using hypoglossal nerve stimulation in sleep apnoea. A total of 46 patients would need enter this two-treatment parallel-design study. This sample size achieves 90% power to detect a clinically important difference of at least 12.3 units (h^-1^) between the null hypothesis that both group means are 32.0 (h^-1^) and the alternative hypothesis that the mean of the trial treatment group is 19.7 (h^-1^) with estimated group standard deviations of 11.8 (h^-1^) and with a significance level of 5%.These calculations were based on 1000 Monte Carlo samples from normal distributions and using a two-sided Mann-Whitney Test. To adjust for the unknown distribution of the primary outcome and based on the lower bound for the asymptotic relative efficiency of the Mann-Whitney U test, we have increased the sample size by a further 15% to 54. Considering our experience with previous studies over this time period we need to account for dropouts and loss-to-follow up of between 15-20%. We therefore propose to study a total sample size of 68, 34 in each group, patients for this study.

*Statistical Methods*

Statistical analysis will compare the respective outcome parameters (primary outcome: AHI; secondary outcome: ESS, 4%ODI, compliance in hours usage/night, comfort) between active stimulation and the control group. Full statistical description will be provided and for each of the variables analysed, univariate descriptive statistics will present an overview of the data. Continuous variables will be presented as median and interquartile range (IQR), unless otherwise stated. For categorical variables, frequency counts and percentages will be presented as summary statistics for the subgroups of interest. To compare study groups, we will use the Wilcoxon and paired t-test for continuous paired variables, and the χ^2^ test for categorical variables. The primary outcome parameters (AHI) will be analysed using the Mann-Whitney test between treatment groups (active stimulation) and control group (CPAP). Similar analyses will be conducted for secondary outcomes. The trial follows up each patient for at least 12 weeks and some patients will be inevitably lost to follow-up. Sample size estimation assumed 20% of patients would not provide end of study information that could be evaluated. If this rate is observed, data for many patients will be only partially observed. For patients who withdraw or drop out before the end of the study, the “no change assumption” will be used to impute the missing subsequent values. This may introduce a bias if the main reason for drop-out was deterioration. To examine this possibility, sensitivity analysis will be performed to assess the primary efficacy outcome using different imputation methods including “best-case scenario”, and “worst- case scenario” possible scores for the missing data as well as Markov Chain Monte Carlo Multiple Imputation. A second per protocol analysis, including only those subjects with complete follow-up data will also be performed. All analyses will be performed using StataTM version 12. Differences will be considered significant at p<0.05.

# Data Management

## Data to be collected

Direct access will be granted to authorised representatives from the sponsor, host institution and the regulatory authorities to permit trial-related monitoring, audits and inspections.

## Data handling and record keeping

The participants will be identified by a study specific participants number and/or code in any database. The name and any other identifying detail will NOT be included in any study data electronic file. The research team will be adhering to Data Protection Act 1998. The data will be collected by the clinical research fellow and controlled by the CI/PI on site, as well as in the quarterly data management meetings with the trial statistician. Records will be kept and archived for 10 years in the Lane Fox Unit CRF.

# Deviations from clinical investigation plan

A deviation is considered a departure from the conditions and principles of GCP in connection with that trial; or the CIP relating to that trial, as amended from time to time. The Investigator shall not deviate from this CIP except in situations that affect the subject’s rights, safety and well-being, or the scientific integrity of the clinical investigation. All CIP deviations shall be recorded and reported to the sponsor as agreed. A deviation log shall be maintained by the study site. Deviations shall be reported to the REC and the regulatory authorities if required by national regulations. All deviations will be included, as required in the final study report.

# Device accountability

The Investigators conducting studies in which investigational devices will be used must follow regulations for device storage, accountability, dispensing/administration and record-keeping.

Investigators will demonstrate understanding of the handling and control of investigational test articles by reviewing this policy and developing a written plan or procedure for ensuring these regulations are met.

*Receipt and Inventory of Study Devices*

This section applies to those study devices the investigator dispenses/administers to the study subject. The investigator (or designated research team member) is responsible for ensuring that the following sections are fully documented and complete. Upon receipt (preferably within 2 working days, but definitely prior to dispensing) of the study device:

- Conduct an inventory of the shipment to ensure that the information on the packing slip matches exactly with what has been sent to the site, to include the receipt date, lot numbers, device type, batch number, code mark, and quantity.

- Identify and document the name of the person who received the shipment of devices as well as their role on the protocol.

- Discrepancies must be promptly (usually within 2-3 working days) reported to the Sponsor/Supplier of the device.

- Documentation of this shipment inventory including the above information must be maintained by the principal investigator or designee.

*Documentation for the Regulatory Binder*

Copies of the following documents must be retained:

- The shipping inventory, packing slips and document inventory in the regulatory binder.
- Accountability log (most Sponsors will issue/supply a device accountability log).

*Labelling of study devices*

Study devices are pre-labeled.

- Do not re-label, deface, or change in any way without prior written permission of the Sponsor.

- It is highly recommended that an additional label may be placed on the study device or packaging, if space and the design of the device permits, to include the study staff contact name/number, but ONLY if the Sponsor agrees in writing prior to doing so.

*Principal Investigator Responsibilities*

The Principal Investigator must be aware of applicable regulations and at a minimum, include the following on the label (if allowed by the sponsor) or on the packaging:

• Name of device
• Model number
• Serial number
• Manufacturer name
• Study staff contact name/number

*Storage of Study Devices*

The Principal Investigator is responsible for establishing and maintaining appropriate access controls for essential and appropriate research personnel. The access controls must include, at a minimum, the following areas:

• Develop procedures for verifying physical access
• Store the study device in a secure environment to include locks on doors and controlled access.
• Establish equipment control both into and out of the research site.
• Develop Security Incident Procedures to report any privacy breaches
• Assess any privacy risks anticipated and develop methods to avoid those risks
• Develop data backup, storage, and emergency mode procedures, if applicable.

• Ensure the study device is stored at the appropriate temperature, and maintain a storage area temperature log, if appropriate.

*Study Device Dispensing*

- The Principal Investigator shall permit an investigational device to be used only with subjects under their personal supervision or under the supervision of a properly trained sub-investigator responsible to the Principal Investigator.

- The Principal Investigator shall not permit an investigational device to be used with subjects who are not under their personal supervision or not under the supervision of a properly trained sub-investigator responsible to the principal investigator.

*Documentation of Study Device Use*

The investigator must create and maintain an access log to document the following:

- Each time the study device is dispensed/used
- Where it is dispensed/used to
- Whom it is dispensed/used
- And the date and signature or initials of the person dispensing/using the study device (plus any other information dictated by the study protocol).

*Documentation of Return/Destruction of the Study Device*

- The Principal Investigator must verify:

- All documentation regarding receipt, storage, dispensing, return of used

containers, and accountability is complete and accurate.

- An explanation of why and how many device units have been returned to the

sponsor, repaired, or otherwise disposed of must he noted. When a device is

disposed of, the identification of the person who doing so must also be noted.

- Devices obtained from a Sponsor for the specific purpose of a research study must be returned to Sponsor. Only with the written authorization (i.e. in the protocol or other written correspondence) of the Sponsor may the investigator discard the device on site, or retain the device.
- Unused study devices that include individually identifiable health information must not be transferred to other investigators without sponsor approval or an authorization from the study subject.
- Unused study devices without individually identifiable health information must not be transferred to other investigators, used for animal research, or dispensed to non-study patients unless written consent is obtained from the Sponsor/Provider of the device.

# Statements of compliance

## Declaration of Helsinki, International Standards and national regulations

The clinical investigation shall be conducted in accordance with the ethical principles of the Declaration of Helsinki, ISO 14155 and all other applicable device and UK regulations.

## Approvals

### The clinical investigation shall not commence recruitment until all REC, Health Research Authority (HRA), other regulatory (if applicable) and confirmation of local capacity and capability is received. All additional requirements imposed by the REC, HRA or regulatory authority will be followed.

## Funding and Insurance

NHS bodies are legally liable for the negligent acts and omissions of their employees. If any patients are harmed whilst taking part in a clinical trial as a result of negligence on the part of a member of the study team this liability cover would apply.

Non-negligent harm is not covered by the NHS indemnity scheme. Guy’s and St Thomas’ NHS Trust, therefore, cannot agree in advance to pay compensation in these circumstances. In exceptional circumstances an ex-gratia payment may be offered. However, legislation now requires any GMC registered medical professional to hold a valid indemnity cover.

# Monitoring

There will be quarterly meetings of the trial steering committee (Chief Investigator, Principal Investigators and Clinical Research Fellow) as well as Data Monitoring meetings every 6-months (Chief Investigator, Statisticians, Clinical Research Fellow).

Case Report Forms (CRFs) will be completed by the investigator and/or his/her delegates within 1 working days of the study visit. All data from the examinations and investigations listed in section 7.4 will be transferred to media provided by the sponsor and collected at the time of CRF collection.

The sponsor will perform interim monitoring. The sponsor will schedule interim monitoring visits with advance notice and confirm the scheduling of the visit with the study site. The sponsor representative should meet with the investigator at each monitoring visit.

The investigator(s) / institution(s) will permit trial-related monitoring, audits, REC review, and regulatory inspection(s), providing direct access to source data/documents. Trial participants are informed of this during the informed consent discussion. Participants will consent to provide access to their medical notes.

The sponsor representative shall perform the following activities at each monitoring visit;

- 10% of source verification
- Essential document review
- Deviation review
- Adverse event review

At the conclusion of the monitoring visit, the sponsor representative shall write a monitoring report detailing the activities performed during the visit with recommendations for action items and study site action. A follow-up letter to the study site detailing these recommendations and actions will be sent to the investigator.

## Confidentiality

All data will be handled in accordance with the UK Data Protection Act 1998. The CRFs will not bear the subject’s name or other personal identifiable data. The subject’s initials, date of birth and trial identification number, will be used for identification. Subjects will be assigned a trial identification number by the study site sequentially starting with 1 upon enrolment into the study. The study site will maintain a master Subject Identification Log.

## Record keeping and archiving

Archiving will be authorised by the Sponsor following submission of the end of study report. Chief Investigators are responsible for the secure archiving of essential trial documents as per their trust policy. All essential documents will be archived for at least 5 years after completion of trial. Destruction of essential documents will require authorisation from the Sponsor.

# Adverse events, adverse device effects and device deficiencies

## Definitions

| **Term** | **Definition** |
| --- | --- |
| **Adverse Event (AE)** | Any untoward medical occurrence, unintended disease or injury, or untoward clinical signs (including abnormal laboratory findings) in subjects, users or other persons, whether or not related to the investigational medical device.  **Note 1:** This definition includes events related to the investigational medical device or the comparator  **Note 2:** This definition includes events related to the procedures involved  **Note 3:** For users or other persons, this definition is restricted to events related to investigational medical devices |
| **Adverse Device Effect (ADE)** | Adverse Event related to the use of an investigational device.  **Note 1:** This definition includes AEs resulting from insufficient or inadequate instructions for use, deployment, implantation, installation, or operation, or any malfunction of the investigational device  **Note 2:** This definition includes any event resulting from use error or from intentional misuse of the investigational medical device |
| **Serious Adverse Event (SAE)** | Any adverse event that:   - Led to death, - Led to serious deterioration in the health of the subject, that either resulted in - a life-threatening illness or injury, or - a permanent impairment of a body structure or a body function, or - in-patient or prolonged hospitalisation, or - medical or surgical intervention to prevent life-threatening illness or injury or permanent impairment to a body structure or a body function, - Led to foetal distress, foetal death or a congenital anomaly or birth defect   **Note 1:** Planned hospitalisation for a pre-existing condition, or a procedure required by the CIP, without serious deterioration in health is not considered a SAE. |
| **Serious Adverse Device Effect (SADE)** | An ADE that has resulted in any of the consequences characteristic of an SAE |
| **Unanticipated Serious Adverse Device Effect (USADE)** | An SADE which by its nature, incidence, severity or outcome has not been identified in the current version of the risk analysis report. |
| **Device Deficiency (DD)** | Inadequacy of a medical device with respect to its identity, quality, durability, reliability, safety or performance.  **Note 1:** this includes malfunctions, use errors, and inadequate labelling |

- An adverse event does not include:
- Medical or surgical procedures; the condition that leads to the procedure is an adverse event.
- Pre-existing disease, conditions, or laboratory abnormalities present at the start of the study that do not worsen in frequency or intensity.
- The disease being studied or signs/symptoms associated with the disease unless more severe than expected for the subject’s condition.

## Safety Reporting requirements and timelines

| **Term** | **Reporter** | **Reported to** | **Reporting Timeline from awareness of the event** |
| --- | --- | --- | --- |
| Adverse Event (AE) | Investigator | Sponsor | As agreed with sponsor |
| Adverse Device Effect (ADE) | Investigator | Sponsor/Manufacturer | As agreed with sponsor |
| Serious Adverse Event (SAE)/ Serious Adverse Device Effect (SADE) | Investigator | Sponsor | Immediately, no more than 24hrs of becoming aware of the event |
|  | Investigator/Sponsor | MHRA[[1]](#footnote-2) | within 2 calendar days of becoming aware of the event. Updates within 7 calendar day. |
|  | Investigator/Sponsor | MHRA Adverse Incident Centre | On CE marked devices |

| Term | Reporter | Reported to | Reporting Timeline from awareness of the event |
| --- | --- | --- | --- |
| Unanticipated Serious Adverse Device Effect (USADE) | Investigator | Sponsor | Immediately, no more than 24 hours |
|  | CI/Sponsor | MHRA/REC | Within 15 calendar days of the CI becoming aware of the event |
| Urgent Safety Measures (USM) | CI/Sponsor | REC | 1. Immediately-By telephone 2. Within 3 days-Notice in writing setting out reasons for the USM and plan for further action |
| Device Deficiency (DD) | Investigator | Sponsor | Immediately, no more than 24 hours of becoming aware of the event |
|  | Sponsor | NCA | 7 calendar days  Only reportable if the event may have led to an SAE if;   - suitable action had not taken - intervention had not been made - if circumstances had been less fortunate |

## Assessments of adverse events

Each adverse event will be assessed for the following criteria:

### Severity

| **Category** | **Definition** |
| --- | --- |
| Mild | The adverse event does not interfere with the subjects daily routine, and does not require intervention; it causes slight discomfort |
| Moderate | The adverse event interferes with some aspects of the subjects routine, or requires intervention, but is not damaging to health; it causes moderate discomfort |
| Severe | The adverse event results in alteration, discomfort or disability which is clearly damaging to health  Note: A severity rating of severe does not necessarily categorise the event as an SAE. |

### Seriousness

Seriousness as defined for an SAE in section 14.1.

### Causality

The assessment of relationship of adverse events to the study procedure and the investigational device will be a clinical decision based on all available information at the time of the completion of the case report form. The following categories will be used to define the causality of the adverse event:

| **Category** | **Definition** |
| --- | --- |
| Yes | There is evidence to suggest a causal relationship, and the influence of other factors is unlikely |
| Possibly | There is some evidence to suggest a causal relationship (e.g. the event occurred within a reasonable time after procedure). However, the influence of other factors may have contributed to the event (e.g. the patient’s clinical condition, other concomitant events). |
| No | There is no evidence of any causal relationship. |

### Expectedness

| **Category** | **Definition** |
| --- | --- |
| *Expected* | An adverse event that is consistent with the information about the device listed in the Investigator Brochure or clearly defined in this CIP. |
| *Unexpected* | An adverse event that is not consistent with the information about the device listed in the Investigator Brochure |

The reference document to be used to assess expectedness against the intervention is the IB. The CIP will be used as the reference document to assess disease related and/or procedural expected events.

## Procedures for recording and reporting Adverse Events and Device Deficiencies

### Investigator responsibilities:

All AEs and SAEs will be recorded in the medical records and CRF following consent.

All SAEs will need to be reported to the sponsor on a SAE form unless stated in the CIP that some expected SAEs will not be reported to the sponsor, with a justification as to why they will not be reported.

For patients on the control arm of a trial, SAEs may not have to be reported to the sponsor but will be recorded in the CRF and medical records.

The Chief or Principal Investigator will complete the sponsor’s serious adverse event form and the form will be emailed to the sponsor R&D@gstt.nhs.uk immediately (or within 24 hours but certainly no later than 24 hours) of his / her becoming aware of the event. The Chief or Principal Investigator will respond to any SAE queries raised by the sponsor as soon as possible

You may choose not to report EXPECTED SAEs to the sponsor for example if they are expected to occur on a regular basis and offer no further new information to your safety profile. These events must continue to be recorded in the source data and CRF, however you may state that you will not complete an SAE form and forward it to the sponsor.

All deaths will be reported to the sponsor irrespective of whether the death is related to disease progression, the device, or an unrelated event. These reports will be provided immediately (or within 24hours) upon notification.

The investigator shall keep detailed records of all adverse events and device deficiencies relating to the clinical trial which are reported to them by trial participants or users. The investigator shall document all relevant information on sponsor provided AE logs, SAE forms and DD forms.

All SAEs, SADEs and USADEs should be reported to R&D@gstt.nhs.uk

## Reporting of all Adverse Events and Device Deficiencies: Investigator and Sponsor responsibilities

Investigator responsibilities shall be as per section 14.4. The sponsor shall keep detailed records of all adverse events and device deficiencies relating to the clinical trial which are reported to them by the trial investigators. The sponsor shall ensure that all relevant information about a reportable event which occurs during the course of this clinical trial in the United Kingdom is reported as soon as possible to the MHRA, and the relevant ethics committees per their reporting requirements and according to the timelines in section 14.2. Any additional relevant information should be sent within the same time frame as the initial report. The Sponsor is responsible for informing the appropriate regulatory authorities, ethics committees and other investigators of any reportable events that have occurred with the study device in any clinical investigation according to the guidelines set forth by either the REC of record or regulatory authority in the country where the clinical investigation is taking place.

### Annual Safety Reports

There is no requirement for an additional Annual Safety Report to the REC or MHRA.

### Progress reports

Progress reports will be submitted to the REC as per the REC requirements. The chief investigator will prepare the progress reports.

## Foreseeable adverse events and anticipated adverse device effects

Transcutaneous electrical neuro-stimulation (TENS) has been used for many years and its effects are well established. Potential adverse events could include:

- skin irritation
- pain

However, having tested this method over >10 years, including a previous randomised controlled trial (TESLA trial, published 2016 in THORAX) we have not experienced any severe adverse events. TENS machines are considered to be safe in use for labour related pain in pregnant women.

# Vulnerable population

No vulnerable population will be selected for recruitment into this trial.

# Suspension or premature termination of the clinical investigation

Each participant has the right to withdraw from the study at any time without providing a reason. In addition, the investigator may discontinue the study at any time if considered necessary for one of the following reasons:

- Ineligibility (either arising during the study or retrospectively),
- Disease progression which requires discontinuation of the study or inability to continue to comply with study procedures,
- Consent withdrawn,
- Lost to follow up.
- The reason for withdrawal will be recorded, including ‘no reason given’, in the case report forms (CRF).

Premature termination of the clinical investigation will be reported to the relevant authorities.

# Publication policy

The data used from the study will be published in an agreed international and peer-reviewed journal by the research team. The data may also be presented at relevant conferences in poster or spoken form as agreed.

# Bibliography

1. Young T, Palta M, Dempsey J, Skatrud J, Weber S, Badr S. The occurence of sleep-disordered breathing among middle-aged adults. N Engl J Med. 1993;328:1230-5.

2. Peppard PE, Young T, Barnet JH, Palta M, Hagen EW, Hla KM. Increased prevalence of sleep-disordered breathing in adults. Am J Epidemiol. 2013;177(9):1006-15.

3. Steier J, Martin A, Harris J, Jarrold I, Pugh D, Williams A. Predicted relative prevalence estimates for obstructive sleep apnoea and the associated healthcare provision. Thorax. 2014;69(4):390-2.

4. Sullivan CE, Issa FG, Berthon-Jones M, Eves L. Reversal of obstructive sleep apnea by continuous positive airway pressure applied through the nares. Lancet. 1981;1:862-5.

5. NICE Technology Appraisal Guidance (TA139). CPAP for OSA costing template and report. 2008. www.nice.org.uk/guidance/ta139

6. Chai-Coetzer CL, Luo YM, Antic NA, Zhang XL, Chen BY, He QY, et al. Predictors of long-term adherence to continuous positive airway pressure therapy in patients with obstructive sleep apnea and cardiovascular disease in the SAVE study. Sleep. 2013;36(12):1929-37.

7. Strollo PJJ, Soose RJ, Maurer JT, de Vries N, Cornelius J, Froymovich O, et al. Upper-airway stimulation for obstructive sleep apnea. New Engl J Med. 2014;370(2):139-49.

8. Steier J, Seymour J, Rafferty GF, Jolley CJ, Solomon E, Luo YM, et al. Continuous transcutaneous submental electrical stimulation in obstructive sleep apnea: a feasibility study. Chest. 2011;140(4):998-1007.

9. Pengo MF, Xiao S, Ratneswaran C, Reed K, Shah N, Chen T, et al. Randomised sham-controlled trial of transcutaneous electrical stimulation in obstructive sleep apnoea. Thorax. 2016;published online first doi:10.1136/thoraxjnl-2016-208691.

10. Pengo MF, Steier J. Emerging technology: electrical stimulation in obstructive sleep apnoea. J Thorac Dis. 2015;7(8):1286-97.

11. Craig SE, Kohler M, Nicoll D, Bratton DJ, Nunn A, Davies R, et al. Continuous positive airway pressure improves sleepiness but not calculated vascular risk in patients with minimally symptomatic obstructive sleep apnoea: the MOSAIC randomised controlled trial. Thorax. 2012;67(12):1090-6.

12. Campbell T, Pengo MF, Steier J. Patients' preference of established and emerging treatments for obstructive sleep apnoea. J Thorac Dis. 2015;7(5):938-42.

13. Randerath WJ, Verbraecken J, Andreas S, et al. Non-CPAP therapies in obstructive sleep apnoea. Eur Respir J. 2011;37(5):1000-28.

14. Eastwood PR, Barnes M, Walsh JH, Maddison KJ, Hee G, Schwartz AR, et al. Treating obstructive sleep apnea with hypoglossal nerve stimulation. Sleep 2011;34(11):1479-86.

15. Schwartz AR, Barnes M, Hillman D, Malhotra A, Kezirian E, Smith PL, et al. Acute upper airway responses to hypoglossal nerve stimulation during sleep in obstructive sleep apnea. Am J Respir Crit Care Med. 2012;185(4):420-6.

16. Strollo PJJ, Malhotra A. Stimulating therapy for obstructive sleep apnoea. Thorax. 2016;published online first doi:10.1136/thoraxjnl-2016-209077.

|  | ***Screening*** | ***Baseline ^a^*** | ***Treatment Phase ^b^*** | | | ***Follow Up ^c^*** |
| --- | --- | --- | --- | --- | --- | --- |
| *Visit / Contact #* | *Visit 1* | *Visit 2* | *Contact 3-7* | *Visit 3* | *Contact 9-13* | *Visit 4* |
|  | *Day – 0* | *Day-1* | *Bi-Weekly follow up phone calls (week 1-5)* | *Follow up Week 6* | *Bi-Weekly follow up phone calls (week 7-11)* | *Follow up Week 12* |
| *Informed Consent* | *X* |  |  |  |  |  |
| *Medical History/Physical exam* | *X* | *X* |  | *X* |  | *X* |
| *Vital Signs* | *X* | *X* |  | *X* |  | *X* |
| *Eligibility determination* |  | *X* |  |  |  |  |
| *Randomisation ^d^* |  | *X* |  |  |  |  |
| *Demographics* |  | *X* |  | *X* |  | *X* |
| *Upper airway anatomy* |  | *X* |  |  |  | *X* |
| *Blood tests* |  | *X* |  |  |  | *X* |
| *Arterial blood gas analysis* |  | *X* |  |  |  | *X* |
| *Polysomnography* |  | *X* |  |  |  | *X* |
| *Symptom scores and quality of life* |  | *X* | *X* | *X* | *X* | *X* |
| *Nocturnal pulse oximetry* |  |  |  | *X* |  |  |
| *Device usage and compliance* |  |  |  | *X* |  | *X* |
| *Adverse Events Review* |  | *X* | *X* | *X* | *X* | *X* |
| *Concomitant Medication review* |  | *X* |  | *X* |  | *X* |
| *Physician’s Withdrawal Checklist* |  | *X* | *X* | *X* | *X* | *X* |

1. SAEs indicating immediate risk of death, serious injury or serious illness and that require prompt remedial action for other patients/subjects, users or other persons or a new finding to it [↑](#footnote-ref-2)
